# Comprehensive lipoprotein and glycoprotein characterization in rheumatoid arthritis plasma and association with clinical markers

**DOI:** 10.3389/fmolb.2025.1627273

**Published:** 2025-09-04

**Authors:** Konstantina Ismini Tsezou, Mohan Ghorasaini, Aswin Verhoeven, Aikaterini Iliou, Dimitra Benaki, Panayiotis G. Vlachoyiannopoulos, Martin Giera, Emmanuel Mikros

**Affiliations:** ^1^ Pathophysiology Department, Medical School, National and Kapodistrian University of Athens, Athens, Greece; ^2^ Pharmagnose S.A., Inofyta, Greece; ^3^ Leiden University Medical Center (LUMC), Center for Proteomics and Metabolomics, Leiden, Netherlands; ^4^ Pharmaceutical Chemistry, Pharmacy School, National and Kapodistrian University of Athens, Athens, Greece; ^5^ Pharma-Informatics Unit, Athena Research and Innovation Center in Information Communication and Knowledge Technologies, Marousi, Greece

**Keywords:** rheumatoid arthritis, disease activity, VAS, triglycerides, HDL, LDL, N-acetylglycoproteins

## Abstract

**Introduction:**

Rheumatoid arthritis (RA) is an autoimmune disease characterized by chronic inflammation and pain. This study investigates plasma lipoprotein and glycoprotein profiles in RA patients to identify clinically relevant markers for disease monitoring.

**Methods:**

Lipoprotein composition and subfractions were analyzed in plasma from 161 RA patients and 46 controls using proton nuclear magnetic resonance (^1^H NMR) spectroscopy (Lipoprotein Subclass Analysis (B.I.LISA) platform) along with N-acetylglycoprotein signals GlycA and GlycB. Lipoprotein subclasses and glycoproteins in RA and disease-modifying anti-rheumatic drug (DMARD)-naive RA patients were compared to controls, and comprehensive profiles were evaluated in activity and remission. Correlations with disease activity score (DAS28), inflammation marker C-reactive protein (CRP), and Visual Analogue Scale (VAS) of pain were assessed using regression models, adjusting for age, gender, and CVD.

**Results:**

RA patients exhibited a distinct lipoprotein and glycoprotein profile, with increased triglycerides, cholesterol, apolipoproteins (A1, A2, B100), and changes in LDL, HDL, GlycA, and GlycB. Glycoproteins were significantly higher in DMARD-naive RA (AUC ≈ 0.9) validating these NMR signals as biomarkers of inflammation. Patients in remission had higher small dense HDL and lower LDL-triglycerides than those with high disease activity. VAS correlated with LDL-triglycerides, while DAS28 correlated with small dense LDL-triglycerides and glycoproteins, inversely with large LDL, small HDL lipids. H4A1 alone characterizes RA remission (AUC ≈ 0.8).

**Conclusion:**

Lipoprotein profiles in RA correlate with disease activity, inflammation, and pain. Large HDL, intermediate LDL and glycoproteins serve for RA monitoring as well as potential molecular markers of pain.

## 1 Introduction

Rheumatoid arthritis (RA) is a severe chronic autoimmune disease, characterized by persistent inflammation, chronic pain, and progressive joint destruction, which manifests locally in the synovium of small joints, but also systemically (Scott et al., 2010; Smolen et al., 2018). The prevalence of RA is up to 1% of the worldwide population, showing a clear female predilection, and commonly presenting after the sixth decade of life with a high heterogeneity in clinical symptoms (Smolen et al., 2018). The diagnosis is based on the ACR/EULAR 2010 classification criteria which involve clinical examination and laboratory results, including autoantibodies, the RA-specific anti-citrullinated protein antibody (ACPA) and Rheumatoid factor (RF) (Kay and Upchurch, 2012). Disease monitoring is currently achieved with several disease activity assessment tools, acute phase reactants as inflammation markers, and physician’s clinical evaluation. Disease Activity Score (DAS28) is a widely used valuable resource which allows to objectively evaluate a patient’s response to treatment, and defines activity stages into Remission, Low, Moderate and High based on the cut-off values (Wells et al., 2008). DAS28 score classification incorporates the following criteria: the number of tender and swollen joints (out of 28), the acute phase reactants erythrocyte sedimentation rate (ESR) or C-reactive protein (CRP) and the global health assessment.

The impact of chronic inflammation in RA is not limited to the small joints but also exerts systemic effects. Early studies have reported perturbations in the metabolic and lipid profile in RA and since then this topic has been thoroughly investigated, in blood, urine and synovial fluid, using a variety of analytical techniques (Xu et al., 2022). Untreated or insufficiently treated RA patients (chronic inflammation) exhibit altered lipid metabolism, particularly lower lipid levels (total Chol and LDL-Chol), a phenomenon known as the lipid paradox (Yan et al., 2023). Despite the observation of lower lipid levels, the risk of developing cardiovascular disease (CVD) remains 2 to 3-fold higher compared to the general population, a condition which is recognized as the leading cause of mortality in RA (Dijkshoorn et al., 2022). This phenomenon cannot be solely attributed to traditional risk factors such as hypertension, smoking and type 2 diabetes but also to the excessive inflammatory burden (Dijkshoorn et al., 2022). Interestingly, RA patients with low levels of inflammation, present with an altered lipid profile, accelerated atherosclerosis and increased CVD risk (Yan et al., 2023).

In the past decade, metabolomics techniques have been applied to investigate unique metabolic fingerprints in RA populations and identify markers that have diagnostic and prognostic capacity. Metabolomics in RA research has been conducted for multiple purposes including i) the differentiation of RA from other diseases, ii) the investigation of the pathophysiological mechanisms in RA initiation and progression, iii) the identification of biomarkers for early diagnosis of disease, iv) the characterization of disease activity status, and v) the monitoring of treatment efficacy. Previous metabolomic studies in RA have identified a spectrum of metabolic alterations, including elevated concentrations of lactate, glutamine, and branched-chain amino acids, alongside reductions in citrate and lipid intermediates, reflecting dysregulation of energy metabolism and immune cell activity (Xu et al., 2022). Additionally, short-chain fatty acids such as butyrate and acetate have been implicated in gut microbiota disturbances and immune modulation (Panfili et al., 2020). Lipidomic investigations have reported significant alterations in phosphatidylcholines, sphingomyelins, and cholesterol esters, many of which correlate with systemic inflammation and cardiovascular comorbidity (Koh et al., 2022; Giera et al., 2012; Duarte-Delgado et al., 2025). These insights have primarily emerged from untargeted NMR and mass spectrometry platforms, which, while powerful for broad metabolic screening, often lack subclass-level resolution and clinical standardization. In contrast, the present study employs targeted ^1^H NMR spectroscopy to quantify lipoprotein subclass composition and circulating glycoproteins, offering detailed insights into lipid carrier remodeling in RA. The Bruker IVDr B.I.LISA platform facilitates reproducible, high-throughput quantification of lipoprotein particle number, size, and lipid content. This platform provides a composition-specific and clinically translatable approach that builds upon and advances existing metabolomic research in RA.

Recently, lipidomics, a subfield of metabolomics that studies the entity of all lipid molecules within biological systems has emerged. Lipids are recognized to play an important role in many disorders, and rapid advances in mass spectrometry-based technologies have enabled their identification and quantitation (Géhin et al., 2023). Moreover, typical laboratory examinations are solely informative of the cholesterol content in blood (total and in individual lipoproteins), as well as total triglycerides, whereas the use of the targeted platform IVDr B.I.LISA on 1D ^1^H NMR spectra allows for the comprehensive quantification of lipid contents within lipoproteins and the assessment of the lipoprotein particle number and size profile (Lodge et al., 2021; Jiménez et al., 2018).

This study aims to describe the distinct lipoprotein profile in active and remission RA population, in an effort to globally map the effect of inflammation and disease activity on the metabolome and determine the association of lipoproteins and their subclasses with clinical parameters currently in use to monitor RA patients, including DAS28, acute phase reactant CRP, but also Visual Analogue Scale of pain (VAS). Additionally, we assess the relation between lipoproteins in RA and the recently introduced markers of systemic inflammation, N-acetylglycoproteins (GlycA and GlycB), identified with ^1^H-NMR (Fuertes-Martín et al., 2020). Lastly, we aim to challenge the performance of the obtained biomarkers in terms of their predictive capacity and their transferability to a clinical setting.

## 2 Methods

### 2.1 Patient enrolment and sample collection and pre-processing

RA patients and healthy volunteers were recruited as part of ArthritisHeal project (No.812890) – Greek population (ArthritisHealGR) at General Hospital of Athens “Laikon” in the Rheumatological outpatient clinic. The collection of human body fluids from healthy and RA patients was approved by the Bioethics and Deontology Committee of the Medical School of the National and Kapodistrian University of Athens (study protocol 296/21-05-2020), as well as the Scientific Council of the General Hospital of Athens, “Laikon” (study protocol 8330/19-05-2020). All participants signed a written informed consent form prior to enrolment and the general data protection regulations, and the Helsinki Declaration were complied with. Blood samples from 161 RA patients, fulfilling the ACR/EULAR criteria at diagnosis, were collected pre-prandially in the morning into sterile, K2 ethylenediaminetetraacetic acid-containing (K-EDTA) tubes (BD, Vacutainer), following a 12 h overnight fast (Kay and Upchurch, 2012). Plasma samples were obtained after low-speed centrifugation at 1,500 *g* for 10 min at 4 °C and were stored at −80 °C within 30 min of collection, where they remained until required for analysis. Information about the study participants including age, gender, autoantibody status (serological markers), acute phase reactants, current therapy and comorbidities were reported in the patients’ medical records. A control population of 46 individuals exhibiting similar prevalence of CVD were recruited as volunteers after being provided with the study details. Patient exclusion criteria include those under 18 years of age, coexisting malignancy, other inflammatory autoimmune disease, and recent hospitalization. RA patients included were naïve to therapy, or receiving DMARDs, biological DMARDs or a combination of both. The index of clinical activity was evaluated by DAS28 joint count using CRP (DAS28-CRP), and RA patients were classified as Remission (DAS28 < 2.6, n = 33), Low (2.6 ≤ DAS28 ≤ 3.2, n = 41), Moderate (3.2 < DAS28 ≤ 5.1, n = 66) and High (DAS28 > 5.1, n = 21). Demographic characteristics and clinical data of the 46 control individuals and the 161 RA patients, classified based on their DAS28 score, are presented in Table 1.

**TABLE 1:** Patient characteristics, including age, gender, auto-antibody status, acute-phase protein levels, therapies and comorbidities. Patients were receiving mono- or combination therapies.

Clinical parameter	Control n = 46	Remission n = 33	Low n = 41	Moderate n = 66	High n = 21
Age (Mean ± SD, Years)	58.2 ± 16.2	52.6 ± 14.4	60.6 ± 10.9	59.6 ± 14.5	66.6 ± 11.4
Gender (F/M)	27/19	23/10	30/11	54/12	18/3
RF +	-	70%	62%	52%	70%
ACPA +	-	65%	55%	49%	78%
CRP (Mean ± SD, mg/L)	-	1.8 ± 2.2	2.3 ± 2.8	7.6 ± 8.8	17.9 ± 17.8
ESR (Mean ± SD, mm/hr)	-	9.6 ± 7	16.2 ± 9.2	25.3 ± 14.7	48.9 ± 23.9
VAS (0–10 cm)	-	2.0 ± 1.2	3.7 ± 1.7	5.3 ± 2.5	7.5 ± 2.4
Therapies					
MTX	-	50%	51%	32%	19%
Glucocorticoids	-	22%	41%	44%	29%
Anti-TNF	-	38%	29%	23%	33%
Anti-IL-6R	-	9%	22%	14%	14%
Anti-CD20	-	9%	12%	8%	14%
Jak inhibitors	-	21%	8%	7%	0%
Comorbidities					
CVD	55%	32%	51%	55%	65%
Thyroid	9%	23%	24%	15%	14%
DM	12%	10%	12%	17%	15%

RF: rheumatoid factor, ACPA: Anti-citrullinated protein antibodies, CRP: C-reactive protein, ESR: erythrocyte sedimentation rate, VAS: visual analogue scale, MTX: methotrexate, Anti-TNF: tumor necrosis factor inhibitor, Anti-IL-6R: Interleukin-6, receptor antibody, anti-CD20: CD20 antibody, Jak inhibitors: Janus kinase inhibitors, CVD: cardiovascular disease, DM: diabetes mellitus.

### 2.2 Sample preparation for IVDr

Sample preparation and quantification of lipoproteins and lipoprotein subclasses was performed according to the requirements of the Bruker IVDr lipoprotein analysis protocol (B.I.LISA^TM^; Lipoprotein Subclass Analysis | Cardiovascular Risk Assessment | Bruker) by NMR spectroscopy, as described in our previous study (Ghorasaini et al., 2022). Briefly, 300 µL of EDTA plasma samples were mixed with 300 µL of 75 mM disodium phosphate buffer in H_2_O/D_2_O (80/20) with a pH of 7.4 containing 6.15 mM NaN_3_ and 4.64 mM sodium 3-[trimethylsilyl] d4-propionate using Gilson 215 liquid handler in combination with Bruker SampleTrack system. Next, the samples were transferred into 5-mm SampleJet NMR tubes in 96 tube racks with a modified second Gilson 215 liquid handling robot. Finally, the tubes were closed by POM ball insertion. While queuing for acquisition, the samples were kept at 6 °C on a SampleJet sample changer.

### 2.3 IVDr experiments for lipoprotein subclass quantification and data processing

A 600 MHz Bruker Avance Neo spectrometer (Bruker Corporation, Billerica, USA) was used to perform ^1^H-NMR experiments. The spectrometer was equipped with a 5-mm triple resonance inverse (TCI) cryogenic probe head complemented by a Z-gradient system and automatic tuning and matching (Findeisen et al., 2021). The NMR spectra were acquired following the Bruker B.I.Methods protocol. Temperature calibration was done before the experiments using a standard 3 mm sample of 99.8% methanol-d_4_ (Bruker). All experiments were recorded at 310 K. A standard 3 mm QuantRefC sample (Bruker) was measured as the quantification reference and for quality control. The duration of the π/2 pulses were calibrated automatically for all individual samples with a homonuclear-gated nutation experiment on the locked and shimmed samples (Wu and Otting, 2005). For water suppression during the relaxation delay and the mixing time of the NOESY1D experiment, pre-saturation of the water resonance with an effective field of γB_1_ = 25 Hz was applied (Price, 1999). The NOESY1D experiment was recorded using the first increment of a NOESY pulse sequence (Kumar, 2003). Using a relaxation delay of 4 s and a mixing time of 10 milliseconds, 32 scans of 98,304 points covering a sweep width of 17,857 Hz were recorded after applying four dummy scans. The NOESY1D spectra were submitted to the commercial Bruker IVDr B.I.-LISA platform to perform regression experiments and extract the concentration values for 112 lipoproteins.

### 2.4 N-acetylglycoprotein and supramolecular phospholipid composite signals measurement

In addition to the targeted NOESY1D spectra acquired for the lipoprotein subclass quantification, 1H 1D T1-edited spectrum using the longitudinal eddy-current delay diffusion (LED) experiment were obtained for the visualization of macromolecular signals profiling. The LED pulse sequence reduces the intensities of small molecules resulting in a spectrum mainly consisting of broad protein signals and thus enabling the detection of protein (lipoprotein, glycoprotein) and lipid moiety alterations. The conversion into numerical data, and the reduction of the number of variables from the full resolution of LED NMR spectra was achieved by bucketing using MATLAB environment (R2021b Mathworks Inc., Natwick, MA, USA). A spectral bucketing of 0.005 was applied at the range of 5.70–0.50 ppm, resulting in 933 spectral bins after removal of water region. Spectral annotation of N-acetylglycoproteins, supramolecular phospholipid composite signals (SPC) and other lipid moieties was performed following previously published work from Nicholson group who carried out 2D NMR experiments and model phospholipid titrations into plasma (Lodge et al., 2021; Masuda et al., 2023). The observed acetyl group resonances correspond to N-acetylglucosamine and N-acetylgalactosamine (GlycA) and N-acetylneuraminic acid (GlycB), while SPC is a composite signal of choline headgroups of lysophosphatidylcholines carried on plasma glycoproteins and from phospholipids in HDL and LDL subfractions. The characteristic N-acetylglycoprotein signals were detected at range of δ = 2.045–2.115; particularly GlycA resonated at δ = 2.045–2.08, with the highest peak at δ = 2.06, and GlycB resonated at δ = 2.085–2.115, with the highest peak at δ = 2.10. The SPC distinguishing signal was represented in the LED spectrum at δ = 3.20–3.30. Lastly, the ratio of SPC/GlycA was calculated from the sum of SPC peaks (δ = 3.20–3.30) and the highest GlycA peak (δ = 2.06), as it is proposed to be a useful marker (Lodge et al., 2021).

### 2.5 Statistical analysis

Due to the complexity of metabolomic and lipidomic data, statistical evaluation was performed with both univariate and multivariate analyses to investigate distinct lipoprotein features in the RA population in high activity and in remission.

The normality of the variables was assessed using the Anderson-Darling test, the D'Agostino and Pearson test, and the Shapiro-Wilk test. Outlier detection and removal was performed with the ROUT method, which is based on nonlinear regression. To account for the potential confounding effects of age, sex, and cardiovascular disease (CVD), we applied a custom MATLAB function (correctForConfounders) that performs linear regression on each lipoprotein and glycoprotein variable. The function estimates the linear contribution of the confounders and subtracts it from each feature, retaining the mean-centered residuals to preserve the original scale. The corrected dataset was subsequently used for all univariate analyses. In pair-wise comparisons, unpaired t-test were applied for parametric data, and Mann-Whitney test for non-parametric data. Volcano plots were constructed to highlight the variable fold-change and statistical significance. Multiple comparisons were achieved with ordinary one-way analysis of variance (ANOVA) and Kruskal-Wallis test for parametric and non-parametric data distribution, respectively. To account for the multiple testing issue, false discovery rate (FDR)-corrected *p*-values were computed. The diagnostic ability (sensitivity and specificity) of markers highlighted from multivariate and univariate analyses, were assessed with receiver operating characteristic (ROC) curve and the corresponding area under the curve (AUC). Univariate analyses were performed with Graphpad Prism 8 (GraphPad Software, Inc.) and MetaboAnalyst 5.0. Statistical power was calculated using SigmaPlot v.14.0 (Systat Software, Inc.).

Multivariate analysis was applied to explore the unique comprehensive lipoprotein profile in RA patients with varying disease activity. Supervised multivariate analysis methods were employed to obtain optimized separations, and account for large inter-group heterogeneity observed in human studies, which are introduced by diverse lifestyle, dietary habits, medication, and other factors. Orthogonal Projections to Latent Structures Discriminant Analysis (OPLS-DA) was used for group comparison to eliminate inter-group variation, hence improve sample classification, and identify possible biomarkers. The normality of the data distribution was assessed and log_10_ transformation was applied when the data were non-parametrically distributed. Data were scaled to the square root of the standard deviation (Pareto) prior to multivariate modelling. The reliability of generated multivariate models is determined by R2 and Q2 parameters, which reflect the descriptive and predictive ability of the model, respectively. To evaluate the accuracy of the OPLS-DA classification and validate the model, permutation testing was performed, calculating 100 random changes. The relevant quality parameters were calculated and compared to those of the original model. The S-plot was used to select important variables in the OPLS-DA analysis, where the covariance (x-axis) and correlation (y-axis) show the contribution and confidence of contribution, respectively, for each variable in the observed classification. Variables with VIP values (Variable importance in projection) > 1.5 were used as a threshold for the selection of the most characteristic in the respective class. Multivariate analyses were performed with SIMCA-P 17.0 (Umetrics, Umea, Sweden) and MetaboAnalyst 5.0. Statistical power was calculated using SigmaPlot v. (Systat Software, Inc.).

Partial correlation was exploited in order to investigate the association of lipoproteins and glycoproteins with the clinical markers: DAS28, CRP and ESR measurements and VAS of pain, following adjustment for age, gender and presence of CVD, using the *spcor.test* function in RStudio (RStudio 2022.07.0, RStudio Team, Boston, MA, USA). The correlation coefficients along with the respective two-tailed *p*-values were calculated and subjected to FDR correction. Furthermore, to evaluate the performance of the comprehensive lipoprotein profile of all RA subjects to predict DAS28, partial least squares (PLS) regression model was constructed and R^2^ was calculated. Lastly, the association of LED spectral bins, which include N-acetylglycoprotein and lipid signals, with clinical parameters was plotted in a pseudospectrum by adapting a statistical heterospectroscopy routine (SHY) in MATLAB environment (Crockford et al., 2006).

## 3 Results

The studied population consisted of 161 patients with RA, stratified by disease activity into remission (n = 33), low (n = 41), moderate (n = 66), and high activity (n = 21) groups, alongside 46 healthy controls (Table 1). The mean age ranged from 52.6 to 66.6 years across RA subgroups, with a female predominance observed in all groups. Clinical and laboratory parameters, including acute-phase reactants CRP and ESR, and pain score, VAS, increased proportionally with disease activity, reflecting the inflammatory burden. The proportion of seropositive individuals (ACPA and/or RF positive) was highest in the high activity group. Patients were receiving various treatment regimens, including conventional synthetic DMARDs, glucocorticoids, biologic agents, and JAK inhibitors. Comorbidities such as cardiovascular disease, diabetes, and thyroid disease were also present at varying frequencies across groups.

In this study, the application of IVDr B.I.LISA method exploited ^1^H NMR data to quantify a total of 112 lipoprotein species, summarized in Supplementary Table S1. An indicative association between lipoprotein size and density is depicted in Supplementary Figure S1. In lipoproteins, size and density exhibit an inverse relationship. Larger lipoproteins, such as chylomicrons and very-low-density lipoproteins (VLDL), are characterized by lower density due to a high triglyceride-to-protein ratio. As lipoproteins decrease in size—progressing through intermediate-density lipoproteins (IDL) to low-density lipoproteins (LDL) and ultimately to high-density lipoproteins (HDL)—they undergo a compositional shift, with a reduction in triglyceride content and an increase in protein proportion, which results in a higher density. Thus, as triglycerides are hydrolyzed and removed from the lipoprotein core, lipoproteins transition to smaller, denser forms (Feingold, 2000).

### 3.1 The lipoprotein profile can differentiate RA from controls

**FIGURE 1:**
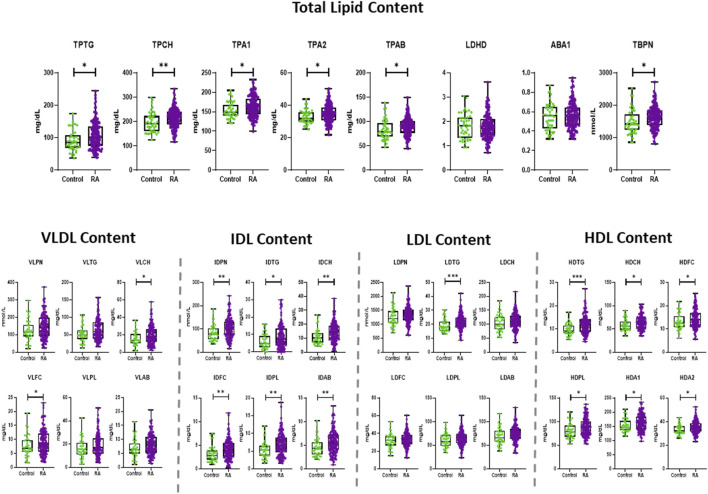
Boxplots showing the distribution and statistical significance (*p*-value) of total lipid contents and lipoprotein concentrations in RA and control, presenting the mean, standard deviation (SD) and minimum and maximum points. The statistical significance is determined by unpaired two-tailed t-test/Mann-Whitney test. Significance is demonstrated with asterisks (*). All lipoprotein species are measured in mg/dL, except particle number (PN), which are measured in nmol/L. *p < 0.05, **p < 0.01, ***p < 0.001.

The comparison of the lipoprotein profiles in plasma of RA individuals and controls showed significant differences regarding total lipoproteins, lipoprotein composition and size abundance. The concentrations and statistical significance of all lipoprotein measurements are presented in Table 2 while changes in distribution are shown in boxplots in Figure 1 and Supplementary Figure S2. The same data, following correction for potential confounding factors, age, gender and CVD, are displayed in Supplementary Table S2.

**TABLE 2:** The concentrations of lipoprotein subclasses in RA and control, presented with the mean and standard deviation (SD).

Total lipid content	Ctr		RA		*p*-value
	Mean	SD	Mean	SD	
TPTG	97.71	46.34	117.27	65.31	2.51E-02
TPCH	196.39	40.20	214.28	39.58	8.20E-03
TPA1	153.26	20.52	163.27	26.05	1.83E-02
TPA2	32.57	4.22	34.75	5.57	1.57E-02
TPAB	82.84	20.16	89.99	18.58	2.63E-02
LDHD	1.81	0.52	1.78	0.50	6.42E-01
ABA1	0.55	0.14	0.56	0.13	5.63E-01
TBPN	1506.24	366.65	1636.19	337.85	1.01E-02
IDL Content					
IDPN	82.96	34.86	103.51	46.31	4.10E-03
IDTG	7.00	6.89	9.68	9.16	1.36E-02
IDCH	11.16	5.60	14.48	7.54	3.20E-03
IDFC	3.21	1.68	4.14	2.23	4.90E-03
IDPL	5.59	2.93	7.06	3.51	1.20E-03
IDAB	4.56	1.92	5.69	2.55	4.10E-03
VLDL Content					
VLPN	130.42	69.70	152.20	81.96	7.54E-02
VLTG	59.86	35.25	72.67	48.02	7.27E-02
VLCH	17.18	11.02	20.80	13.30	2.36E-02
VLFC	7.92	4.42	9.42	5.44	4.20E-02
VLPL	17.07	9.36	19.69	11.04	9.06E-02
VLAB	7.17	3.83	8.37	4.51	7.61E-02
V1TG	22.86	19.44	29.58	31.66	2.54E-01
V2TG	9.59	7.33	11.52	7.72	1.65E-02
V3TG	9.50	6.73	11.44	6.91	1.98E-02
V4TG	8.47	4.18	10.31	4.84	6.50E-03
V5TG	2.69	0.83	3.02	0.98	4.59E-02
V1CH	4.81	3.91	6.04	6.50	3.01E-01
V2CH	2.59	2.03	3.08	2.18	3.15E-02
V3CH	3.19	2.44	3.98	2.72	1.88E-02
V4CH	4.77	2.85	6.00	3.04	6.50E-03
V5CH	1.40	0.73	1.44	0.79	7.19E-01
V1FC	1.68	1.60	2.09	2.23	3.07E-01
V2FC	1.08	0.92	1.32	1.12	6.22E-02
V3FC	1.31	1.08	1.67	1.31	1.51E-02
V4FC	1.89	1.28	2.52	1.59	1.09E-02
V5FC	0.61	0.43	0.72	0.65	4.88E-01
V1PL	3.99	3.19	4.82	4.59	5.32E-01
V2PL	2.65	1.88	3.09	1.94	2.14E-02
V3PL	3.30	2.23	3.95	2.35	3.33E-02
V4PL	4.33	2.11	5.18	2.39	2.04E-02
V5PL	1.68	0.82	1.77	0.85	5.49E-01
LDL Content					
LDPN	1248.08	326.90	1331.32	301.51	1.10E-01
LDTG	19.25	4.61	21.86	5.48	6.00E-04
LDCH	105.71	30.92	111.33	29.24	2.62E-01
LDFC	31.60	8.14	33.16	8.22	2.59E-01
LDPL	61.01	14.92	63.92	14.54	2.38E-01
LDAB	68.64	17.98	73.22	16.58	1.10E-01
L1PN	234.72	62.62	265.81	71.48	8.80E-03
L2PN	162.46	71.44	173.52	67.89	3.41E-01
L3PN	164.37	67.01	169.94	68.61	6.29E-01
L4PN	155.72	74.39	146.34	74.41	4.56E-01
L5PN	190.29	78.78	192.99	69.77	8.24E-01
L6PN	338.72	123.61	386.29	116.74	3.60E-03
L1TG	6.20	2.02	7.28	2.49	4.20E-03
L2TG	2.45	0.73	2.75	0.80	1.90E-03
L3TG	2.34	0.67	2.62	0.75	2.18E-02
L4TG	2.21	0.97	2.26	0.97	6.06E-01
L5TG	2.42	0.84	2.56	0.89	3.51E-01
L6TG	3.94	1.08	4.60	1.35	5.00E-04
L1CH	23.84	7.19	26.63	7.99	3.43E-02
L2CH	15.82	8.30	16.84	7.58	4.36E-01
L3CH	15.06	7.17	15.47	7.15	4.52E-01
L4CH	13.26	6.65	12.31	6.56	3.93E-01
L5CH	14.54	6.46	14.33	5.67	8.32E-01
L6CH	22.81	8.32	25.66	7.36	3.80E-03
L1FC	7.45	2.09	8.37	2.35	1.76E-02
L2FC	5.43	2.36	5.96	2.27	1.76E-01
L3FC	5.34	2.05	5.54	2.06	5.65E-01
L4FC	4.46	1.63	4.44	1.63	9.62E-01
L5FC	4.37	1.54	4.44	1.36	3.91E-01
L6FC	5.84	1.89	6.54	1.68	2.90E-03
L1PL	13.77	3.58	15.33	4.08	2.10E-02
L2PL	9.12	4.09	9.65	3.87	4.24E-01
L3PL	8.71	3.52	8.91	3.60	7.46E-01
L4PL	7.70	3.38	7.23	3.39	4.15E-01
L5PL	8.09	3.17	7.93	2.85	7.34E-01
L6PL	12.85	3.99	14.33	3.64	2.60E-03
L1AB	12.91	3.44	14.62	3.93	8.80E-03
L2AB	8.93	3.93	9.54	3.73	3.40E-01
L3AB	9.04	3.69	9.35	3.77	5.22E-01
L4AB	8.56	4.09	8.05	4.09	4.56E-01
L5AB	10.47	4.33	10.61	3.84	8.23E-01
L6AB	18.63	6.80	21.25	6.42	3.60E-03
HDL Content					
HDTG	9.91	3.09	11.92	4.01	2.00E-04
HDCH	59.59	13.78	64.45	14.80	1.72E-02
HDFC	13.06	3.52	14.46	4.10	3.90E-02
HDPL	80.90	16.03	88.56	18.46	1.27E-02
HDA1	152.91	22.36	163.02	27.94	2.31E-02
HDA2	32.97	4.12	35.11	5.39	1.47E-02
H1TG	3.31	1.72	4.48	1.95	3.30E-04
H2TG	1.54	0.63	1.99	0.77	3.98E-04
H3TG	1.93	0.73	2.31	0.83	4.50E-03
H4TG	3.36	1.21	3.45	1.22	6.64E-01
H1CH	18.65	9.76	22.73	9.19	2.90E-03
H2CH	8.25	2.77	9.51	2.80	6.40E-03
H3CH	10.53	1.89	11.47	2.33	1.41E-02
H4CH	21.49	4.04	20.58	4.53	2.27E-01
H1FC	4.42	2.35	5.36	2.26	5.60E-03
H2FC	1.95	0.74	2.32	0.71	2.20E-03
H3FC	2.21	0.53	2.52	0.69	4.80E-03
H4FC	4.12	0.85	4.24	1.08	5.02E-01
H1PL	21.96	11.76	27.27	11.04	1.20E-03
H2PL	12.68	3.91	14.81	3.98	2.90E-03
H3PL	16.73	3.03	18.26	3.55	8.90E-03
H4PL	29.05	4.60	28.28	5.36	1.51E-01
H1A1	26.91	17.03	33.50	15.58	2.90E-03
H2A1	18.86	4.45	21.20	4.91	7.70E-03
H3A1	27.34	4.55	29.71	5.54	9.10E-03
H4A1	79.84	12.01	77.89	13.16	2.02E-01
H1A2	2.49	1.65	3.41	1.56	2.00E-04
H2A2	3.31	1.07	4.10	1.15	5.37E-05
H3A2	6.19	1.26	7.01	1.52	1.30E-03
H4A2	20.19	3.94	19.75	4.04	5.19E-01

The *p*-value was calculated to assess statistical significance. Significant lipoprotein species (*p* < 0.05) are displayed in red-scaled shading. All lipoprotein species are measured in mg/dL, except particle number (PN), which are measured in nmol/L.

RA exhibited significantly elevated concentrations of total plasma triglycerides (TPTG) and plasma cholesterol (TPCH), Apolipoprotein A1 (TPA1), A2 (TPA2) and B100 (TPAB) compared to controls (Figure 1). VLDL subclass concentrations were marginally elevated in RA, particularly in the Chol, free Chol and Triglyceride (TG) content of small and intermediate sized particles (VLDL-2 – VLDL-5), and most evidently in VLDL-4 (Supplementary Figure S2). The IDL plasma levels (IDPN) were overall increased, which was also reflected in the significantly higher concentrations of all its lipid components (Figure 1). The total LDL content in plasma of RA was comparable to that of controls, besides the LDL-TGs (LDTG) concentration which were significantly elevated in RA (Figure 1), as demonstrated in large, but also very small LDL particle sizes (L1TG, L2TG, L3TG, L6TG). Interestingly, the concentration of large (L1PN) and small (L6PN) size LDL particles were selectively higher in RA, as well as all LDL lipid constituents in those subfractions (Supplementary Figure S2). HDL presented the most profound alteration in RA, due to increased concentrations of lipid content, particularly TGs (HDTG), in large sized, less dense particles (H1TG, H2TG, H3TG). This implies that HDL particles with large and intermediate size were significantly elevated (HDL-1, HDL-2, HDL-3), whereas the highest density particles (HDL-4) remained unaltered in the general RA population (Supplementary Figure S2).

**FIGURE 2:**
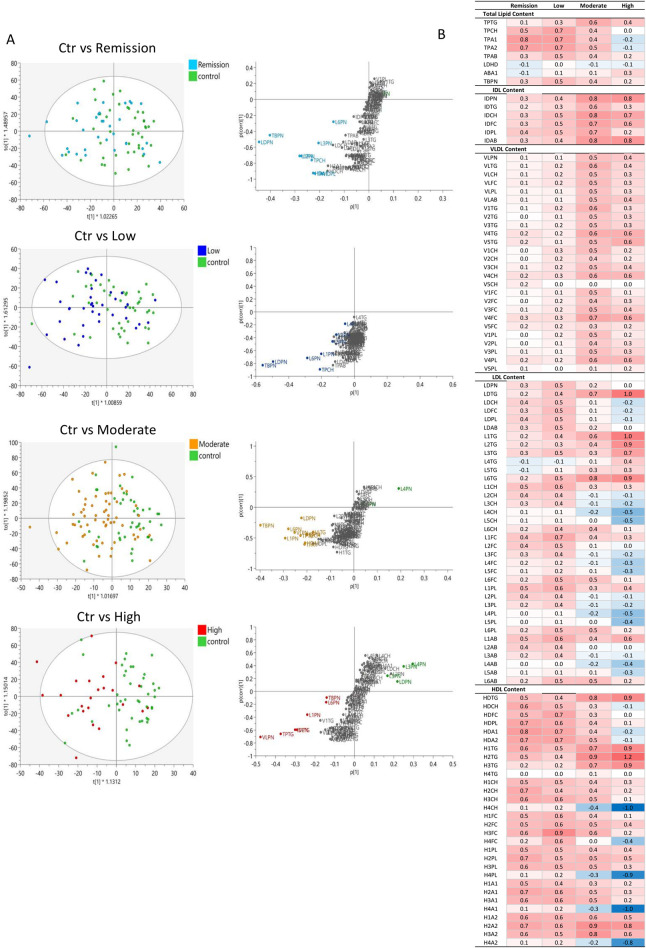
Comparison of the lipoprotein profile of DAS28-classified RA subgroups with controls. **(A)** OPLS-DA scores plots showing the discrimination of lipoprotein profiles in plasma of DAS28-classified RA individuals (Remission (light blue; (R2(cum) = 0.685; Q2(cum) = -0.037), Low (blue; (R2(cum) = 0.629; Q2(cum) = 0.039), Moderate (orange; (R2(cum) = 0.836; Q2(cum) = 0.105), High (red; (R2(cum) = 0.658; Q2(cum) = 0.154) activity RA) versus Controls (green). The corresponding S-plots demonstrate the lipoprotein features significantly elevated in RA subgroups (bottom, left) and Controls (top, right), which are responsible for the clustering (VIP>1.5). **(B)** Heatmap of the z-score of lipoproteins in RA subclasses compared to controls, coloured based on positive (red) and negative (blue) z-score values.

The evaluation of the global lipoprotein profile in RA with multivariate statistical analysis tools demonstrated a moderate discrimination between RA and controls (Figure 2). The OPLS-DA model confirms the findings of the univariate analysis and highlights elevated concentrations of total ApoB carrying (TBPN) and LDL particles (LDPN), specifically LDL-6 and LDL-1 as well as TPTG and TPCH and lower levels of LDL-4 (L4PN). TBPN and LDPN appear as weighted components in the multivariate models likely due to their higher concentrations in plasma, with their distribution varying from 1636.19 ± 337.85 nmol/L and 1331.32 ± 301.51 mg/dL, respectively in RA individuals. This model is reliable, as suggested by the valid permutation test, however it demonstrated a low discriminant and predictive capacity (R2(cum) = 0.759; Q2(cum) = 0.084) perhaps owed to the complexity of the RA population. Therefore, the lipoproteins and lipoprotein subfraction measurements were also examined in DAS28-classified RA patients versus control (Figure 2). The concentrations of the lipoprotein subclasses in the control and RA population with different disease activity is presented in Supplementary Table S3, and the statistically significant features are shown in Supplementary Table S4. Lipoprotein features in the different DAS28-classified RA and controls were also presented in Supplementary Table S5, following adjustment for potential confounding effects of age, sex, and cardiovascular disease (CVD) status. In addition, to assess the influence of varying sample size in our study groups on statistical robustness, a *post hoc* power analysis (α = 0.05) was performed and presented in Supplementary Table S6. Variables demonstrating the strongest statistical significance (lowest *p*-values) also exhibited power values exceeding 0.80, indicating reliable detection of group differences and supporting their robustness, while features with marginal significance (e.g., *p* ≈ 0.05) corresponded to reduced statistical power.

Pairwise comparisons indicated a separation of High and Moderate DAS28 RA groups from controls (Figure 2A). The reliability of the comparative models is evaluated with permutation test in Supplementary Figure S4. The heatmap presenting the z-score of the lipoprotein features in DAS28-classified RA groups compared to controls reveals a common profile between remission and low activity RA groups and moderate and high activity RA groups (Figure 2B). Despite the lack of discrimination between controls and Remission as well as controls and Low activity RA in multivariate models, individual features, particularly HDL, show elevated levels in remission (Figure 2B; Supplementary Table S4). Lipoprotein features in the different DAS28-classified RA and controls are also presented in Supplementary Table S5, following adjustment for potential confounding effects of age, sex, and cardiovascular disease (CVD) status.

### 3.2 The lipoprotein profile distinguishes remission and high disease activity in RA

**FIGURE 3:**
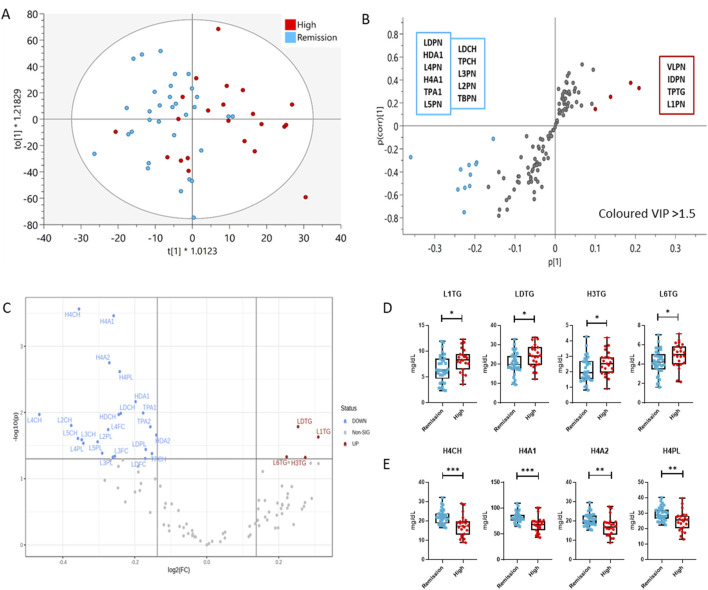
Comparison of the lipoprotein profile of Remission (blue) and High DAS28 RA patients (red) using multivariate and univariate analysis. **(A)** OPLS-DA scores plot showing the discrimination of the lipoprotein profiles of the two groups (R2(cum) = 0.785; Q2(cum) = 0.189), **(B)** S-plot demonstrates the lipoprotein features significantly elevated in Remission (bottom, left) and High DAS28 (top, right), which are responsible for the clustering (VIP>1.5). The features in grey, located centrally, do not significantly impact the separation. **(C)** Volcano plot demonstrates significant changes (1.1-fold change, *p* < 0.05) in lipoprotein subfraction in High DAS28 compared to Remission. **(D)** Boxplots showing the individual values of lipoprotein subclasses increased in High DAS28 RA, and **(E)** those that are increased in Remission. The statistical significance is determined by unpaired two-tailed t-test/Mann-Whitney test. Significance is demonstrated with asterisks (*). *p < 0.05, **p < 0.01, ***p < 0.001.

In addition to the altered lipoprotein profile observed in our study, we investigated the differential lipoprotein total concentration, individual classes and subclasses and variable densities in RA remission and high disease activity. The concentrations of the lipoprotein (sub) classes in the DAS28-classified RA population are presented in Supplementary Table S3, and the statistically significant features are shown in Supplementary Table S4 and Supplementary Table S5. Multivariate analysis resulted in a valid OPLS-DA model with a significant separation and distinct lipoprotein profile in remission compared to high activity RA patients (Figures 3A,B). Patients with high DAS28 were characterized by increased levels of VLDL and IDL particle number (VLPN, IDPN), TPTG and large, less dense LDL particles (L1PN) compared to those in remission. RA individuals in remission exhibited higher total LDL particle number (LDPN), particularly, in intermediate density LDL particles (L2PN, L3PN, L4PN, L5PN) and the Chol component (LDCH). Additionally, total and HDL apolipoprotein A1 (TPA1, HDA1), particularly in small, dense HDL particles (H4A1), are elevated in remission compared to active disease. Fold changes and significance of lipoprotein composition differences between the study groups were visualized with a volcano plot and boxplots (Figures 3C–E). The volcano plot highlighted a 10% increase in LDL TGs (LDTG, L1TG, L6TG) in highly active RA, and reduced small, dense HDL subclasses (H4CH, H4A1, H4A2, H4PL), Apolipoprotein A1 (TPA1, HDA1) and A2 (TPA2, HDA2), Cholesterol (HDCH) as well as LDL-Chol (LDCH), particularly of intermediate density LDL particles (L2CH, L2PL, L3CH, L3FC, L3PL, L4CH, L4PL, L4FC) in remission. The sensitivity and specificity of the aforementioned markers in predicting the disease activity state (high activity or remission) was determined by ROC curves, and the AUC values are presented in Supplementary Table S7. Small, dense HDL particles (H4CH, H4A1, H4A2, H4PL) have the best predictive capacity (AUC > 0.75), particularly H4A1 (AUC = 0.8) is potentially a useful marker in predicting activity or remission in RA.

### 3.3 The lipoprotein profile in RA is associated with disease activity and markers of systemic inflammation

**FIGURE 4:**
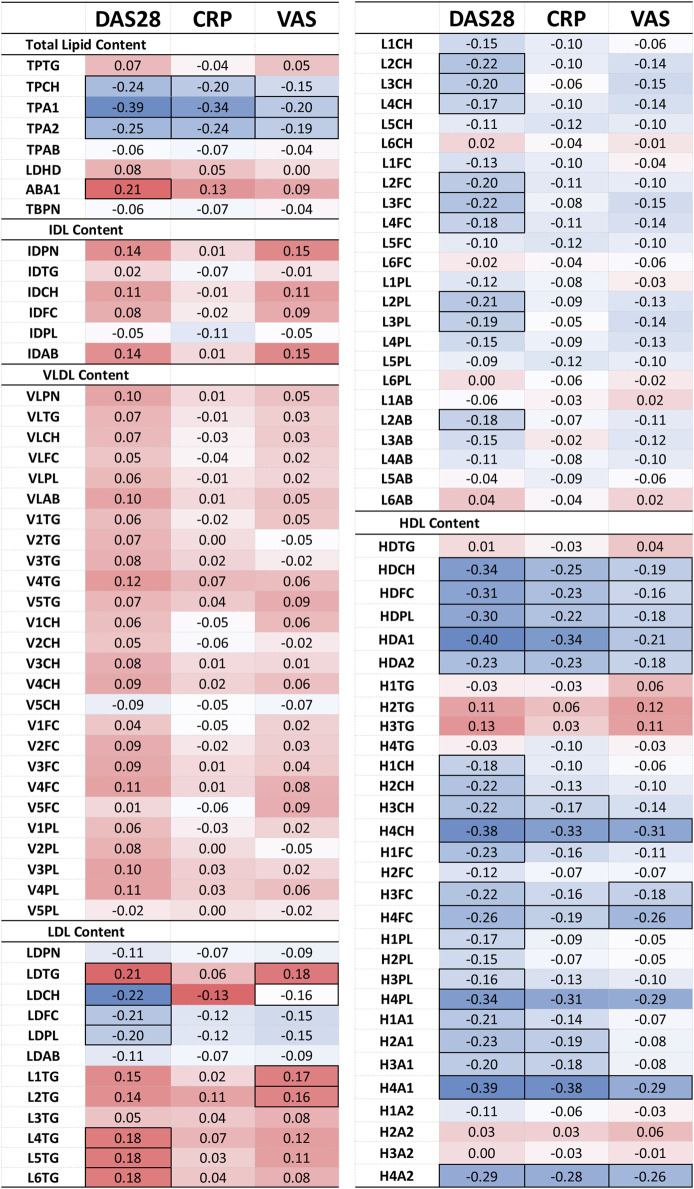
Heatmap of the partial correlation of IVDr lipoprotein subclasses with clinical markers DAS28, CRP, and VAS, following correction for age, gender and CVD presence. The correlation coefficient (r) is coloured based on positive (red) and negative (blue) correlation. Statistically significant correlation based on the *p*-value (*p* < 0.05) are identified by highlighted cell border.

Furthermore, removing the DAS28-classificiation criteria, the comprehensive lipoprotein profile of 161 individuals with RA was associated with the clinical markers DAS28, CRP and VAS used in RA monitoring, following correction for age, gender, and CVD presence. The results are presented in a heatmap in Figure 4 and Supplementary Table S8.

The correlation of lipoprotein component and particle totals with clinical markers was relatively weak (maximum r-corr = −0.40), except for the TPCH which was inversely correlated with all clinical parameters, particularly, DAS28. TPTG showed a weak positive association to DAS28 and VAS, while Apo-B100/Apo-A1 ratio (ABA1) was significantly correlated with DAS28. Apolipoprotein A1 (TPA1) and A2 (TPA2) correlate quite strongly with DAS28 and CRP, but these are HDL-only proteins which will be discussed separately below.

VLDL and IDL (sub) fractions do not show a significant association with any clinical markers, however, LDL and its subfractions demonstrate a distinct pattern of association with the clinical markers. LDL components, Chol (LDCH), Free Chol (LDFC) and Phospholipids (LDPL), inversely correlate with DAS28. More specifically, the large and intermediate LDL particles, LDL-2 and LDL-3, show a distinctive pattern of negative association with DAS28. In contrast, LDL TGs (LDTG) positively correlated with DAS28 (r = 0.21) and VAS (r = 0.18), especially, small, dense LDL (LDL-4, LDL-5, LDL-6) with DAS28 (r = 0.18), and large, less dense LDL TG (L1TG, L2TG) with VAS (r = 0.17).

HDL particles exhibited a more pronounced relation to the clinical parameters. HDL lipid components were strongly inversely associated with all clinical parameters, especially in small, dense HDL (HDL-4) Chol (H4CH), phospholipid (H4PL), apolipoprotein A1 (H4A1) and A2 (H4A2). These lipoproteins demonstrated an inverse relation to the acute phase reactant CRP, indicating that dense HDL particles, especially the Chol and apoA1 subfractions, are decreased in inflammation.

**FIGURE 5:**
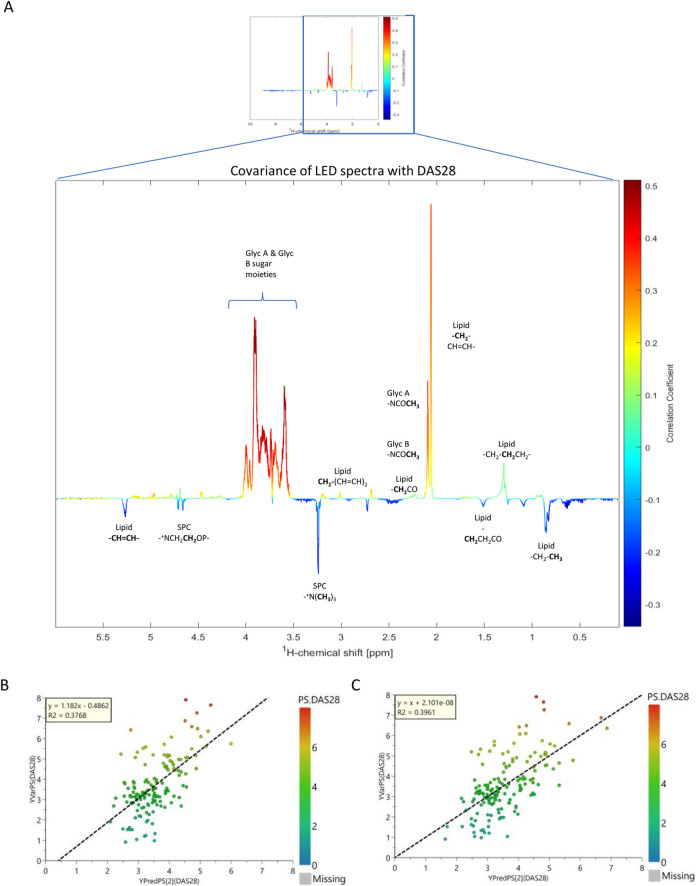
Correlation of lipids with DAS28. **(A)** NMR pseudo-spectrum shows the covariance of the 161 NMR LED spectra, containing 1040 features (lipid signals) with the DAS28 score. The coloring of the peaks is based on the correlation coefficient value (r); red and blue indicate a positive and negative correlation, respectively. **(B)** PLS regression plot of the 120 IVDr lipoprotein and lipoprotein subfraction concentrations, colored by DAS28 score. **(C)** PLS regression plot of the NMR LED 1040 features (lipid signal), colored by DAS28 score. The coefficient of determination (R^2^) is a statistical measure of how accurately the regression predictions fits the data.

A PLS regression model was employed to assess whether the lipoprotein fingerprint of RA patients can predict their disease activity (Figure 5). Indeed, it showed that lipoproteins, following variable selection (VIP>1), were able to modestly predict (R^2^ = 0.37) the DAS28 score (Figures 5B,C).

### 3.4 N-acetylglycoproteins and lipoprotein supramolecular phospholipids correlate with clinical markers in RA

As glycoproteins have previously been linked to many inflammatory conditions, as well as RA (Fuertes-Martín et al., 2020; Rodríguez-Carrio et al., 2020; Dudka et al., 2021), their signal intensity and their relation to RA-monitoring markers were evaluated in our study population. A representative NMR LED spectrum showing N-acetylglycoprotein (GlycA and GlycB) and lipoprotein supramolecular phospholipid composite (SPC) signals was annotated and presented in Supplementary Figure S5 (Lodge et al., 2021). GlycA and GlycB were significantly elevated in RA compared to controls, particularly in moderate and high disease activity populations, whereas the SPC signals were rather elevated in remission (Supplementary Figure S6). Additionally, GlycA and GlycB levels were differentiated in the various disease activity stages (Remission, Low, Moderate, High) of RA, especially between remission/low activity and moderate/high (Supplementary Figure S6; Supplementary Table S9). This result was confirmed in DMARD-naive RA population, demonstrating remarkable predictive capacity (GlycA+B: AUC = 0.90) (Supplementary Figure S7; Supplementary Table S10). Unlike lipoproteins, which had a relatively weak association with clinical markers, the signals obtained from the N-acetylglycoproteins showed a stronger association. Furthermore, GlycA and GlycB positively correlated with DAS28 (r = 0.31; r = 0.29), CRP (r = 0.21; r = 0.18) and VAS (r = 0.21; r = 0.24), following correction for age, gender, and CVD presence (Supplementary Table S11). In contrast, SPC indicated an inverse correlation with CRP (r = −0.23), suggesting a role in reduction of inflammation. These observations are confirmed from the pseudospectrum generated from the statistical correlation of the entire spectral profile with DAS28 (Figure 5A). The SPC/Glyc ratio did not demonstrate a particular association to the examined clinical markers. The covariance between the entire LED spectrum, entailing lipid and glycoprotein features, with clinical markers was examined in NMR pseudospectra, presented in Supplementary Figure S8. All markers showed a strong correlation to GlycA and GlycB signals, and inverse correlation to SPC. DAS28 demonstrated the strongest correlation to glycoproteins (r ≈ 0.5). DAS28 and CRP appeared to be inversely associated with peaks corresponding to lipid species, while VAS exhibited a different pattern.

### 3.5 The significant relation of lipoprotein particles with glycoproteins and cholines

**FIGURE 6:**
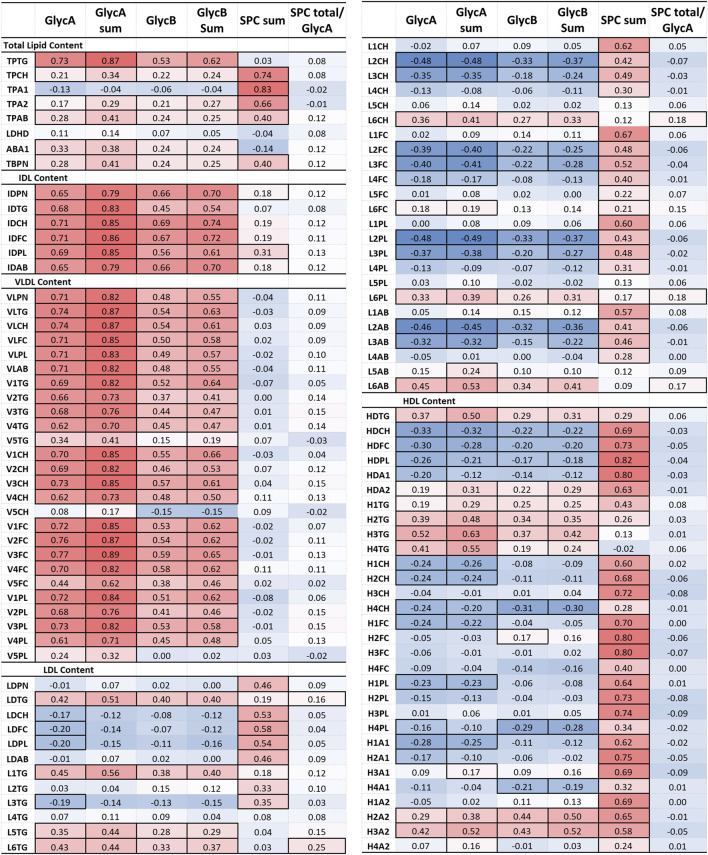
Partial correlation of glycoprotein and SPC signals with IVDr lipoprotein subclasses following correction for age, gender and CVD presence. The correlation coefficient (r) is coloured based on positive (red) and negative (blue) correlation. Statistically significant correlation based on the *p*-value (*p* < 0.05) are identified by highlighted cell border.

Aside from the relation to clinical markers, we investigated whether GlycA and GlycB signals are associated with lipoproteins, following correction for age, gender, and CVD presence, and whether they prove superior markers for disease monitoring. N-acetylglycoprotein NMR signals strongly correlate with the lipoprotein profile of 161 RA patients. Results are shown in heatmaps in Figure 6 and Supplementary Table S12. GlycA and GlycB demonstrated an inverse relation to SPC, as such, when the former were positively associated with a certain lipoprotein, the latter showed a negative association to that. N-Acetylglycoproteins signals, particularly, GlycA sum, demonstrate a remarkable correlation with total TG (r = 0.87), IDL (r ≈ 0.8), VLDL (r ≈ 0.8), and the TG content in large, less dense LDL (L1TG; r = 0.56), small, dense LDL particles (L5TG, L6TG; r = 0.44) and HDL particles (H2TG, r = 0.39; H3TG, r = 0.52; H4TG; r = 0.41). Moreover, they also present modest, but significant, association to TPCH, total apoA2 (TPA2), apoB (TPAB) and apoB-carrying particles (TBPN), as well as ABA1. GlycA and GlycB show a similar pattern of inverse relation to LDL-2 (r ≈ −0.45) and LDL-3 (r ≈ −0.35) particles, as observed with DAS28 GlycA and GlycB show a similar pattern of inverse relation to LDL-2 and LDL-3 particles, and HDL lipid content, except TGs, as observed with DAS28. Evidently, the sum of all GlycA peaks has the highest association with the aforementioned markers and proves to be of greater significance than the highest peak alone (GlycA).

SPC was distinctively associated with total Chol (TPCH, r = 0.74), apoA1 and A2 (TPA1, r = 0.83; TPA2, r = 0.66) and total B-carrying particles (TBPN; r = 0.40), all LDL and HDL lipid subclasses, specifically on small and intermediate sized particles (LDL-1 – LDL-4, r ≈ 0.5; HDL1 – HDL-3, r ≈ 0.7). The ratio of SPC to GlycA exhibited a similar trend to GlycA and GlycB, nevertheless, it only correlated significantly with LDL TGs, and lipid components of LDL-6 particles.

### 3.6 Triglycerides are an objective measure of pain

Pain is an uncomfortable sensation that is subjectively evaluated with a self-reported measure, the VAS score. In this RA population, VAS accurately reflects the disease activity (r = 0.664), and to a lesser extent the inflammatory state (CRP, r = 0.286; ESR, r = 0.343) (Supplementary Figure S9A). While the pain is not directly associated with lipid alterations, we have investigated the relationship between pain severity in RA and lipoproteins. The association of VAS with the lipoprotein profile revealed a similar pattern with DAS28, being also a weighted component. Specifically, VAS was positively correlated with TGs, particularly those of large, less dense LDL particles, and N-acetylglycoprotein signals (Supplementary Tables S8, S12). Additionally, a low VAS score was associated with Chol, apoA1 and apoA2 of small dense HDL particles. To verify the importance of TGs in pain in RA and eliminate possible influence of therapeutic agents, the association of VAS with the lipoprotein profile was investigated in DMARD-naïve RA patients and the results were presented in Supplementary Figure S9B. VAS of DMARD-naïve individuals strongly correlated with large density LDL-TGs (L6TG, r^2^ = 0.62) and HDL-TGs (HDTG, r^2^ = 0.56), particularly on large HDLs (H1TG, r^2^ = 0.59; H2TG, r^2^ = 0.29), and inversely correlated with small, dense HDL (HDL-4) subclasses, including Apolipoprotein A2 (H4A2, r^2^ = 0.46), Chol (H4CH, r^2^ = 0.41), and Phospholipids (H4PL, r^2^ = 0.37).

## 4 Discussion

This study explored the lipoprotein profile in RA, by employing ^1^H NMR-based targeted analysis using Bruker IVDr Lipoprotein Subclass Analysis B.I.LISA platform. We observed a distinct lipoprotein signature in RA compared to control individuals, and the changes mediated by disease activity, inflammatory markers, and pain. Importantly, we determined markers associated with high disease activity and remission and assessed their diagnostic capacity.

NMR spectroscopy was employed for this study considering its high reproducibility, minimal sample preparation, and ability to quantify subclass-specific lipoprotein composition, particle number, and glycoproteins in a single run, offering standardized, high-throughput clinical applications that are particularly suited for population-based studies and biomarker discovery.

The lipid profile of the RA population demonstrated distinct characteristics compared to controls. Perturbations were apparent mainly in intermediate sized HDL and selectively in the largest and smallest LDL particles. In RA, the composition of all lipoproteins was most profoundly increased in TG compared to controls, which progressively increased with disease activity. Previous studies also reported higher serum TG levels and TG-enriched lipoproteins in RA compared to healthy populations (Yan et al., 2023; Arts et al., 2012; Kim et al., 2016).

Furthermore, the investigation of the effect of pain on RA lipid profile showed that VAS correlated with total LDL TGs, particularly of small LDL in RA, as well as large LDL and small HDLs in DMARD-naïve RA individuals, suggesting that TGs measurements can be potentially of clinical relevance for estimating pain and possible treatment options.

In the presence of elevated circulating TGs, TG-rich lipoproteins are formed which become entrapped in the endothelium, and subsequently oxidized (Yan et al., 2023). Oxidized lipoproteins become engulfed by macrophages and form foam cells which exhibit impaired immune functions and contribute to the pathogenesis. Consistent with previous reports, significant reduction in large HDL, HDL2-Chol and HDL3-Chol have been described in RA, which accords to the elevated large and intermediate HDL in our cohort (Arts et al., 2012; Kim et al., 2016; Chang et al., 2023). Previous studies, including those by Arts et al. (2012) and Kim et al. (2016) reported decreased HDL-C levels in RA patients using conventional methods focused on cholesterol content within HDL2 and HDL3 subfractions. However, these methods do not capture lipid compositional changes. In contrast, our study employed a novel application of ^1^H NMR spectroscopy to assess subclass-specific HDL particle concentration and lipid composition, revealing increased cholesterol content in small, dense HDL particles (HDL-4) in RA remission. This reflects inflammation-associated remodeling of HDL particles, resulting in compositional alterations that reduce their cholesterol load while enriching them in triglycerides. These findings offer a complementary, high-resolution perspective on HDL dysfunction in RA not accessible through traditional lipid profiling.

### 4.1 The lipoprotein profile distinguishes remission and high disease activity in RA

Patients with high DAS28 displayed increased levels of VLDL and IDL levels, as well as total TG, particularly in large and small LDL compared to remission. High activity is reflective of an inflammatory state, with a number of affected joints and localized pain. Specifically, DAS28 correlates with TGs in large density LDL particles, and VAS with that in small density LDL particles.

Individuals with RA have a higher CVD risk, partly due to perturbations in the lipoprotein profile (Yan et al., 2023). Elevated circulating TG levels lead to TG enrichment in ApoB-containing lipoproteins. Hepatic lipase-mediated lipolysis of TG-rich, apo C-III-containing remnants generates smaller, denser LDL particles, that more readily penetrate the endothelium, increasing vascular permeability and leukocyte infiltration. These remodeled LDL particles exhibit reduced affinity for LDL receptors, resulting in prolonged circulation and increased susceptibility to oxidative modification. Macrophages engulf oxidized LDL (ox-LDL) via scavenger receptors, forming foam cells that contribute to vascular inflammation. Small, dense LDL also bind to glycosaminoglycans, trapping TG-rich lipoproteins in the subendothelial space (Yan et al., 2023). Additionally, lipolysis of TG-rich lipoproteins generates oxidized fatty acids, that induce endothelial inflammation, insulin resistance, NF-κB activation, and expression of adhesion molecules, contributing to leukocyte recruitment and macrophage cytotoxicity (Yan et al., 2023). These inflammatory processes are accompanied by a dysregulated cytokine milieu, including elevated levels of IL-6, IL-17, and TNF-α, which are hallmarks of RA (Yan et al., 2023; Lei et al., 2023). Altered lipid homeostasis may further influence adaptive immunity by facilitating Th1 and Th17 cell differentiation and activation, thereby perpetuating synovial inflammation (Yan et al., 2023; Lei et al., 2023; Lim et al., 2014). These mechanisms support the relevance of lipid metabolism in RA immunopathogenesis and help contextualize the observed lipoprotein abnormalities.

RA individuals in remission exhibited higher LDL particle number, especially elevated Chol content in medium-sized LDL particles. This is consistent with the recovery of normal lipid levels observed after induction of therapy to inflammation and disease activity (Lei et al., 2023). LDL components (Chol, Free Chol and phospholipids) specifically in intermediate LDL particles portray a unique pattern of inverse correlation with DAS28. A negative association between lipid fractions and disease activity markers, DAS28 and CDAI has been previously reported (Cacciapa et al., 2018).

Additionally, large HDL particles, as well as their individual lipid components ApoA1 are elevated in remission. HDL components strongly inversely correlate with DAS28, CRP and VAS. Evidently, the entire HDL profile, except the TG component, portrays the most significant inverse relationship to DAS28. In addition, high DAS28 RA demonstrated lower HDL levels, particularly of large HDL, and an inverse relation to DAS28, CRP and VAS. Large HDL subclasses potentially serve as useful predictors of RA activity or remission. HDL-Chol levels in RA were previously reported to have an inverse correlation to DAS28 and ESR hence markers of disease remission and systemic inflammation reduction (Arts et al., 2012). This was recently reiterated in a study that showed an inverse association of CRP levels with dense HDLs in RA (Chang et al., 2023). Another study has also demonstrated a protective role of ApoA1, ApoA2 and dense HDL (Lei et al., 2023; Yuan et al., 2023).

HDL particles adapt dynamically to physiological and pathological stimuli. HDL-Chol lowers CVD risk by promoting cholesterol efflux, reducing immune activation, and preventing LDL oxidation (Yan et al., 2023). However, HDL-Chol-raising therapies failed to prevent CVD, as the properties of HDL are strongly linked to its size and composition (Ronsein and Heinecke, 2017). Inflammation reduces HDL levels and alters its composition by depleting cholesterol esters, increasing phospholipase A2 activity, and generating TG-rich, CE-poor HDL, which are rapidly cleared, impairing their protective functions (Yan et al., 2023). Additionally, ApoA1 declines while HDL incorporates inflammatory molecules like amyloid A1 and complement C3, shifting HDL from a cardioprotective to an inflammation-associated role (Grao-Cruces et al., 2022).

Overall, esterified, and free Chol, as well as phospholipids in LDL and HDL particles were inversely related to disease activity and markers of inflammation. Specifically, intermediate LDL particles and large HDL demonstrated a prominent pattern of inverse correlation with the clinical markers. HDL ApoA1 and ApoA2 were highlighted in disease remission and the reduction of inflammation and pain, could potentially indicate a role in RA pathological mechanisms.

### 4.2 N-acetylglycoproteins and lipoprotein supramolecular phospholipids correlate with clinical markers in RA

Growing evidence suggests that glycoproteins serve as key biomarkers of inflammation, as structural changes in proteins alter glycan chains, creating distinct glycoprotein profiles linked to systemic inflammation (Mehta et al., 2020). ^1^H NMR spectroscopy enables the identification and quantification of glycan moieties by detecting signals from N-acetylglucosamine and N-acetylgalactosamine (GlycA), and N-acetylneuraminic acid (GlycB) (Fuertes-Martín et al., 2018). Elevated N-acetyl signals from glycosylated amino sugars have been observed in inflammatory conditions (Lodge et al., 2021; Fuertes-Martín et al., 2020; Rodríguez-Carrio et al., 2020; Iliou et al., 2023).

GlycA and GlycB were significantly elevated in RA compared to controls, and differentiated in the various disease activity stages of RA. The importance of glycoproteins in RA was confirmed in DMARD-naïve population, which demonstrated a remarkable predictive capacity, thus suggesting that elevated GlycA levels are an early event in the course of RA. Elevated GlycA concentrations in RA have been reported by previous cross-sectional studies (Dudka et al., 2021; Ormseth et al., 2015; Bartlett et al., 2016).

Importantly, GlycA and GlycB demonstrated a significant association with DAS28, CRP and VAS, following adjustment for age, gender, and presence of CVD. In contrast, SPC which represents the choline NMR signal derived from bound phospholipids, indicated an inverse correlation with CRP, implying a role in reduction of inflammation and remission. These findings reinforce the results of studies that reported reduced GlycA and GlycB levels in bDMARD therapy responders, hence deeming N-acetylglycoproteins a promising biomarker for assessing effective response to therapy. GlycA, correlates with existing markers in clinical use including DAS28, CRP, ESR, fibrinogen, IL-6, IL-1b and TNF-α levels (Fuertes-Martín et al., 2018; Bartlett et al., 2016; McGarrah et al., 2017). To the best of our knowledge, this is the first report associating N-Acetylglycoproteins with a marker for pain, VAS. While GlycA is a useful indicator of inflammation, it has not been specifically linked to pain itself. However, elevated levels of proteoglycans have been linked to muscle stiffness and contractures in cerebral palsy, which can be perceived as pain (Smith et al., 2021). In addition, GlycA, reflecting α1-acid glycoprotein, is associated to atherosclerosis, plaque vulnerability, and CVD risk (Rodríguez-Carrio et al., 2020; Dudka et al., 2021). This could be of particular significance in RA patients, where traditional markers like LDL-Chol and CRP are not consistent, largely due to the lipid paradox.

### 4.3 The significant relation of lipoproteins particles with glycoproteins and cholines

Furthermore, we investigated the association of GlycA and GlycB signals with lipoprotein lipid content and density, and their potential as markers for disease monitoring. Our study reveals a strong correlation between the peaks resulting from N-acetylglycoprotein signals and the lipoprotein profile of RA patients. GlycA and GlycB exhibit an inverse relation to SPC. As such, when the former were positively associated with a certain lipoprotein, the latter showed a negative association.

GlycA and GlycB demonstrate a strong correlation with IDL, VLDL, and the TG content in small LDL particles and HDL particles. Interestingly, N-acetylglycoproteins demonstrate a stronger inverse correlation with large HDL. The inverse association of HDL-Chol levels with GlycA was also previously reported in individuals with metabolic syndrome (Gruppen et al., 2016). Glycation of HDL promotes aortic stiffness, endothelial dysfunction, and atherosclerosis by impairing LDL clearance and reducing nitric oxide (Ronda et al., 2015). The CATHGEN study linked smaller HDL subclasses to reduced mortality risk, opposing the effects of GlycA (Kim et al., 2016). Furthermore, we demonstrated that the parallel increase in SPC and HDL levels can be explained by the enhanced phosphocholines content in HDL in remission. Therefore, we propose SPC as a useful marker for monitoring inflammation and disease activity and characterizing remission.

The main strength of this study lies in its comprehensive analysis of lipoproteins and apolipoproteins, including their lipid content and the particle size distribution. Unlike previous studies which have primarily focused on total lipids or cholesterol content, our approach provides a more detailed characterization of the lipoprotein profile in RA. By integrating glycoprotein profiling, we offer a novel perspective on systemic inflammation and its metabolic consequences. However, certain limitations should be acknowledged. The control group was not matched to the RA population based on age and gender, but the large, recruited cohort and statistical adjustments were implemented to mitigate this concern. Moreover, the findings were derived from a single RA cohort without an independent validation set, limiting the generalizability of the results and increasing the risk of overfitting, particularly in multivariate models. Second, despite statistical adjustments for demographic factors such as age, gender, and cardiovascular disease, certain subgroups—most notably the high disease activity group—were relatively small, potentially reducing statistical power and affecting the robustness of subgroup comparisons. Nevertheless, power analysis validated statistically significant features in comparisons between high DAS28 individuals and other study groups. Future research should include larger, independent cohorts to confirm these findings and aid in clinical translation, perhaps also employing benchtop strategies, as well as assessing the impact of different treatment strategies on lipoprotein and glycoprotein alterations to better understand their role in RA pathogenesis (Wist et al., 2025).

## 5 Conclusion

This study reveals the strong link between lipoproteins, glycoproteins, and systemic inflammation in RA, showing that an altered lipoprotein profile correlates with disease activity, inflammation, and pain. Specific lipoprotein subfractions, such as small, dense HDL-4 and intermediate-density LDL-2 and LDL-3, help classify RA remission, while GlycA signals, small, dense LDL-6, and TG-rich lipoproteins characterize exacerbated RA. The finding of elevated TG content in lipoproteins in active disease is observational evidence, and the mechanism needs further verification. These findings support lipid and glycoprotein biomarkers for disease monitoring and highlight their role in RA pathophysiology. Further research could clarify their mechanistic links to RA progression and therapeutic potential, improving disease management and patient outcomes. From a technical perspective, these findings could be of great value if translated to a clinical setting using benchtop technologies.

## Data Availability

The data are not publicly available to preserve the privacy of the participants of the LAIKO study. Requests to access the datasets should be directed to the corresponding author.

## Abbreviations

All lipoprotein species have been abbreviated in Supplementary Figure S1. RA: Rheumatoid arthritis, DMARD: disease-modifying anti-rheumatic drug, DAS28: disease activity score, CRP: C-reactive protein, VAS: Visual Analogue Scale, ACPA: anti-citrullinated protein antibody, RF: Rheumatoid factor, GlycA: N-acetylglucosamine and N-acetylgalactosamine, GlycB: N-acetylneuraminic acid, SPC: supramolecular phospholipid composite, ox-LDL: oxidized LDL.
