# Supplementary material for: Comprehensive lipoprotein and glycoprotein characterization in rheumatoid arthritis plasma and association with clinical markers
Source: Front Mol Biosci. 2025 Sep 4;12:1627273. doi: 10.3389/fmolb.2025.1627273 (PMC12443556; doi:10.3389/fmolb.2025.1627273)
Supplement: Supplementary file 1 [file DataSheet1.pdf]

Supplementary Table 1: Abbreviations and full names of lipoprotein particles and their subclasses measured with NMR-based Bruker in vitro diagnostics for research (IVDr) lipoprotein subclass analysis (B.I.LISA) service.

Abbreviation	Class/subclass	Compound	Unit
**TPTG**	Total Plasma	Triglycerides	mg/dL
**TPCH**	Total Plasma	Cholesterol	mg/dL
**LDCH**	LDL	Cholesterol	mg/dL
**HDCH**	HDL	Cholesterol	mg/dL
**TPA1**	Total Plasma	Apolipoprotein-A1	mg/dL
**TPA2**	Total Plasma	Apolipoprotein-A2	mg/dL
**TPAB**	Total Plasma	Apolipoprotein-B100	mg/dL
**LDHD**	Ratio LDL and HDL Cholesterol	LDL Cholesterol / HDL Cholesterol	-
**ABA1**	Ratio of Apolipoproteins A1 and B100	Apolipoprotein-A1/ Apolipoprotein-B100	-
**TBPN**	Apolipoprotein-B100 carrying particles	Particle Number	nmol/L
**VLPN**	VLDL	Particle Number	nmol/L
**IDPN**	IDL	Particle Number	nmol/L
**LDPN**	LDL	Particle Number	nmol/L
**L1PN**	LDL-1	Particle Number	nmol/L
**L2PN**	LDL-2	Particle Number	nmol/L
**L3PN**	LDL-3	Particle Number	nmol/L
**L4PN**	LDL-4	Particle Number	nmol/L
**L5PN**	LDL-5	Particle Number	nmol/L
**L6PN**	LDL-6	Particle Number	nmol/L
**VLTG**	VLDL Class	Triglycerides	mg/dL

Supplementary Table 1 continued

Abbreviation	Class/subclass	Compound	Unit
**IDTG**	IDL Class	Triglycerides	mg/dL
**LDTG**	LDL Class	Triglycerides	mg/dL
**HDTG**	HDL Class	Triglycerides	mg/dL
**VLCH**	VLDL Class	Cholesterol	mg/dL
**IDCH**	IDL Class	Cholesterol	mg/dL
**LDCH**	LDL Class	Cholesterol	mg/dL
**HDCH**	HDL Class	Cholesterol	mg/dL
**VLFC**	VLDL Class	Free Cholesterol	mg/dL
**IDFC**	IDL Class	Free Cholesterol	mg/dL
**LDFC**	LDL Class	Free Cholesterol	mg/dL
**HDFC**	HDL Class	Free Cholesterol	mg/dL
**VLPL**	VLDL Class	Phospholipids	mg/dL
**IDPL**	IDL Class	Phospholipids	mg/dL
**LDPL**	LDL Class	Phospholipids	mg/dL
**HDPL**	HDL Class	Phospholipids	mg/dL
**HDA1**	HDL Class	Apolipoprotein-A1	mg/dL
**HDA2**	HDL Class	Apolipoprotein-A2	mg/dL
**VLAB**	VLDL Class	Apolipoprotein-B100	mg/dL
**IDAB**	IDL Class	Apolipoprotein-B100	mg/dL
**LDAB**	LDL Class	Apolipoprotein-B100	mg/dL
**V1TG**	VLDL-1 Subclass	Triglycerides	mg/dL
**V2TG**	VLDL-2 Subclass	Triglycerides	mg/dL
**V3TG**	VLDL-3 Subclass	Triglycerides	mg/dL
**V4TG**	VLDL-4 Subclass	Triglycerides	mg/dL
**V5TG**	VLDL-5 Subclass	Triglycerides	mg/dL

Supplementary Table 1 continued

Abbreviation	Class/subclass	Compound	Unit
**V1CH**	VLDL-1 Subclass	Cholesterol	mg/dL
**V2CH**	VLDL-2 Subclass	Cholesterol	mg/dL
**V3CH**	VLDL-3 Subclass	Cholesterol	mg/dL
**V4CH**	VLDL-4 Subclass	Cholesterol	mg/dL
**V5CH**	VLDL-5 Subclass	Cholesterol	mg/dL
**V1FC**	VLDL-1 Subclass	Free Cholesterol	mg/dL
**V2FC**	VLDL-2 Subclass	Free Cholesterol	mg/dL
**V3FC**	VLDL-3 Subclass	Free Cholesterol	mg/dL
**V4FC**	VLDL-4 Subclass	Free Cholesterol	mg/dL
**V5FC**	VLDL-5 Subclass	Free Cholesterol	mg/dL
**V1PL**	VLDL-1 Subclass	Phospholipids	mg/dL
**V2PL**	VLDL-2 Subclass	Phospholipids	mg/dL
**V3PL**	VLDL-3 Subclass	Phospholipids	mg/dL
**V4PL**	VLDL-4 Subclass	Phospholipids	mg/dL
**V5PL**	VLDL-5 Subclass	Phospholipids	mg/dL
**L1TG**	LDL-1 Subclass	Triglycerides	mg/dL
**L2TG**	LDL-2 Subclass	Triglycerides	mg/dL
**L3TG**	LDL-3 Subclass	Triglycerides	mg/dL
**L4TG**	LDL-4 Subclass	Triglycerides	mg/dL
**L5TG**	LDL-5 Subclass	Triglycerides	mg/dL
**L6TG**	LDL-6 Subclass	Triglycerides	mg/dL
**L1CH**	LDL-1 Subclass	Cholesterol	mg/dL
**L2CH**	LDL-2 Subclass	Cholesterol	mg/dL
**L3CH**	LDL-3 Subclass	Cholesterol	mg/dL
**L4CH**	LDL-4 Subclass	Cholesterol	mg/dL

Supplementary Table 1 continued

Abbreviation	Class/subclass	Compound	Unit
**L5CH**	LDL-5 Subclass	Cholesterol	mg/dL
**L6CH**	LDL-6 Subclass	Cholesterol	mg/dL
**L1FC**	LDL-1 Subclass	Free Cholesterol	mg/dL
**L2FC**	LDL-2 Subclass	Free Cholesterol	mg/dL
**L3FC**	LDL-3 Subclass	Free Cholesterol	mg/dL
**L4FC**	LDL-4 Subclass	Free Cholesterol	mg/dL
**L5FC**	LDL-5 Subclass	Free Cholesterol	mg/dL
**L6FC**	LDL-6 Subclass	Free Cholesterol	mg/dL
**L1PL**	LDL-1 Subclass	Phospholipids	mg/dL
**L2PL**	LDL-2 Subclass	Phospholipids	mg/dL
**L3PL**	LDL-3 Subclass	Phospholipids	mg/dL
**L4PL**	LDL-4 Subclass	Phospholipids	mg/dL
**L5PL**	LDL-5 Subclass	Phospholipids	mg/dL
**L6PL**	LDL-6 Subclass	Phospholipids	mg/dL
**L1AB**	LDL-1 Subclass	Apolipoprotein-B100	mg/dL
**L2AB**	LDL-2 Subclass	Apolipoprotein-B100	mg/dL
**L3AB**	LDL-3 Subclass	Apolipoprotein-B100	mg/dL
**L4AB**	LDL-4 Subclass	Apolipoprotein-B100	mg/dL
**L5AB**	LDL-5 Subclass	Apolipoprotein-B100	mg/dL
**L6AB**	LDL-6 Subclass	Apolipoprotein-B100	mg/dL
**H1TG**	HDL-1 Subclass	Triglycerides	mg/dL
**H2TG**	HDL-2 Subclass	Triglycerides	mg/dL
**H3TG**	HDL-3 Subclass	Triglycerides	mg/dL
**H4TG**	HDL-4 Subclass	Triglycerides	mg/dL
**H1CH**	HDL-1 Subclass	Cholesterol	mg/dL

Supplementary Table 1 continued

Abbreviation	Class/subclass	Compound	Unit
**H2CH**	HDL-2 Subclass	Cholesterol	mg/dL
**H3CH**	HDL-3 Subclass	Cholesterol	mg/dL
**H4CH**	HDL-4 Subclass	Cholesterol	mg/dL
**H1FC**	HDL-1 Subclass	Free Cholesterol	mg/dL
**H2FC**	HDL-2 Subclass	Free Cholesterol	mg/dL
**H3FC**	HDL-3 Subclass	Free Cholesterol	mg/dL
**H4FC**	HDL-4 Subclass	Free Cholesterol	mg/dL
**H1PL**	HDL-1 Subclass	Phospholipids	mg/dL
**H2PL**	HDL-2 Subclass	Phospholipids	mg/dL
**H3PL**	HDL-3 Subclass	Phospholipids	mg/dL
**H4PL**	HDL-4 Subclass	Phospholipids	mg/dL
**H1A1**	HDL-1 Subclass	Apolipoprotein-A1	mg/dL
**H2A1**	HDL-2 Subclass	Apolipoprotein-A1	mg/dL
**H3A1**	HDL-3 Subclass	Apolipoprotein-A1	mg/dL
**H4A1**	HDL-4 Subclass	Apolipoprotein-A1	mg/dL
**H1A2**	HDL-1 Subclass	Apolipoprotein-A2	mg/dL
**H2A2**	HDL-2 Subclass	Apolipoprotein-A2	mg/dL
**H3A2**	HDL-3 Subclass	Apolipoprotein-A2	mg/dL
**H4A2**	HDL-4 Subclass	Apolipoprotein-A2	mg/dL

Supplementary Table 2: The concentrations of lipoprotein subclasses in RA (n=161) and control (n=46), presented with the mean and standard deviation (SD), following correction for confounding factors age, gender and CVD. Unpaired t-test was performed to assess statistical significance, and the *p*-value was calculated. Significant lipoprotein species ($p < 0.05$) are displayed in red-scaled shading. All lipoprotein species are measured in mg/dL, except particle number (PN), which are measured in nmol/L.

	Ctr		RA		
	**Mean**	**SD**	**Mean**	**SD**	***p*-value**
**Total Lipid Content**					
TPTG	117.31	64.57	97.71	43.84	1.02E-02
TPCH	213.39	35.77	196.39	39.63	6.10E-03
TPA1	163.16	22.97	153.26	17.75	8.10E-03
TPA2	34.66	5.27	32.57	3.96	1.09E-02
TPAB	89.61	17.66	82.84	19.84	1.22E-02
LDHD	1.77	0.47	1.81	0.48	5.38E-01
ABA1	0.56	0.13	0.55	0.13	6.35E-01
TBPN	1629.44	321.17	1506.24	360.81	8.40E-03
**IDL Content**					
IDPN	102.70	45.89	82.96	31.16	5.30E-03
IDTG	9.69	9.06	7.00	6.64	1.24E-02
IDCH	14.33	7.46	11.16	5.24	6.60E-03
IDFC	4.09	2.20	3.21	1.59	6.60E-03
IDPL	7.01	3.49	5.59	2.91	1.40E-03
IDAB	5.65	2.52	4.56	1.71	7.40E-03
**VLDL Content**					
VLPN	152.11	80.83	130.42	64.50	5.61E-02
VLTG	72.73	47.26	59.86	33.71	2.79E-02
VLCH	20.69	13.25	17.18	10.60	3.66E-02
VLFC	9.40	5.41	7.92	4.23	4.34E-02
VLPL	19.68	10.92	17.07	8.97	4.79E-02
VLAB	8.37	4.45	7.17	3.55	5.61E-02
V1TG	29.71	30.95	22.86	18.09	1.72E-01
V2TG	11.53	7.63	9.59	7.11	1.43E-02
V3TG	11.43	6.82	9.50	6.51	8.80E-03
V4TG	10.30	4.75	8.47	3.92	2.17E-02
V5TG	3.02	0.95	2.69	0.76	3.76E-02
V1CH	6.02	6.42	4.81	3.70	3.05E-01
V2CH	3.06	2.17	2.59	1.99	5.05E-02
V3CH	3.95	2.71	3.19	2.39	2.34E-02
V4CH	5.95	3.01	4.77	2.70	9.50E-03

Supplementary Table 2 continued

V5CH	1.44	0.78	1.40	0.70	7.31E-01
V1FC	2.08	2.20	1.68	1.54	2.10E-01
V2FC	1.31	1.11	1.08	0.90	7.92E-02
V3FC	1.67	1.30	1.31	1.05	4.08E-02
V4FC	2.50	1.57	1.89	1.20	1.35E-02
V5FC	0.72	0.65	0.61	0.41	4.70E-01
V1PL	4.83	4.50	3.99	3.05	3.79E-01
V2PL	3.08	1.93	2.65	1.83	2.16E-02
V3PL	3.93	2.33	3.30	2.16	1.84E-02
V4PL	5.16	2.37	4.33	1.97	4.12E-02
V5PL	1.77	0.84	1.68	0.79	5.67E-01
**LDL Content**					
LDPN	1325.83	282.25	1248.08	325.14	1.15E-01
LDTG	21.82	5.18	19.25	4.11	3.84E-01
LDCH	110.73	26.81	105.71	30.29	2.75E-01
LDFC	33.01	7.45	31.60	7.96	5.62E-01
LDPL	63.64	13.30	61.01	14.69	2.46E-01
LDAB	72.92	15.52	68.64	17.88	1.15E-01
L1PN	264.33	62.90	234.72	62.29	5.50E-03
L2PN	172.71	63.28	162.46	68.57	3.41E-01
L3PN	169.05	63.61	164.37	63.98	6.82E-01
L4PN	145.08	72.19	155.72	70.27	3.64E-01
L5PN	191.73	68.28	190.29	74.24	9.44E-01
L6PN	385.92	114.62	338.72	122.26	2.90E-03
L1TG	7.27	2.35	6.20	1.77	8.50E-03
L2TG	2.74	0.74	2.45	0.70	4.00E-03
L3TG	2.62	0.69	2.34	0.65	1.61E-02
L4TG	2.25	0.94	2.21	0.90	9.29E-01
L5TG	2.54	0.88	2.42	0.75	6.27E-01
L6TG	4.61	1.27	3.94	1.02	2.00E-04
L1CH	26.43	6.95	23.84	7.16	1.91E-02
L2CH	16.76	7.06	15.82	7.81	4.37E-01
L3CH	15.37	6.63	15.06	6.67	7.91E-01
L4CH	12.20	6.34	13.26	6.29	3.19E-01
L5CH	14.24	5.52	14.54	6.12	7.59E-01
L6CH	25.63	7.21	22.81	8.28	3.20E-03
L1FC	8.31	2.01	7.45	2.08	1.22E-02
L2FC	5.93	2.11	5.43	2.22	6.80E-02
L3FC	5.52	1.89	5.34	1.90	5.62E-01
L4FC	4.42	1.54	4.46	1.57	8.90E-01
L5FC	4.42	1.33	4.37	1.49	8.18E-01
L6FC	6.53	1.65	5.84	1.88	4.00E-03
L1PL	15.24	3.55	13.77	3.57	1.46E-02
L2PL	9.61	3.60	9.12	3.86	4.24E-01

Supplementary Table 2 continued

L3PL	8.86	3.32	8.71	3.30	7.97E-01
L4PL	7.18	3.29	7.70	3.21	3.41E-01
L5PL	7.88	2.80	8.09	2.99	6.64E-01
L6PL	14.33	3.58	12.85	3.97	2.20E-03
L1AB	14.54	3.46	12.91	3.43	5.50E-03
L2AB	9.50	3.48	8.93	3.77	3.40E-01
L3AB	9.30	3.50	9.04	3.52	6.82E-01
L4AB	7.98	3.97	8.56	3.86	3.64E-01
L5AB	10.55	3.76	10.47	4.08	8.97E-01
L6AB	21.22	6.30	18.63	6.72	2.90E-03
**HDL Content**					
HDTG	11.92	3.68	9.91	2.73	5.16E-06
HDCH	64.47	13.40	59.59	11.79	2.38E-02
HDFC	14.48	3.54	13.06	3.14	1.43E-02
HDPL	88.56	15.62	80.90	13.69	4.50E-03
HDA1	162.94	24.69	152.91	19.23	9.20E-03
HDA2	35.02	5.14	32.97	3.91	1.04E-02
H1TG	4.49	1.73	3.31	1.54	5.92E-05
H2TG	1.99	0.70	1.54	0.57	1.18E-04
H3TG	2.30	0.79	1.93	0.66	1.40E-03
H4TG	3.44	1.21	3.36	1.12	6.45E-01
H1CH	22.79	8.36	18.65	8.55	1.40E-03
H2CH	9.51	2.47	8.25	2.42	2.40E-03
H3CH	11.45	2.06	10.53	1.67	5.10E-03
H4CH	20.56	4.51	21.49	3.97	2.14E-01
H1FC	5.36	2.03	4.42	2.08	7.00E-03
H2FC	2.32	0.61	1.95	0.67	6.00E-04
H3FC	2.51	0.60	2.21	0.48	1.50E-03
H4FC	4.23	1.04	4.12	0.83	4.65E-01
H1PL	27.34	9.82	21.96	10.42	8.00E-04
H2PL	14.81	3.37	12.68	3.50	1.20E-03
H3PL	18.23	3.07	16.73	2.79	2.60E-03
H4PL	28.25	5.29	29.05	4.53	2.86E-01
H1A1	33.58	14.04	26.91	15.27	4.30E-03
H2A1	21.22	4.27	18.86	4.02	4.10E-03
H3A1	29.69	4.90	27.34	4.22	6.50E-03
H4A1	77.80	13.04	79.84	11.97	2.27E-01
H1A2	3.42	1.41	2.49	1.51	3.00E-04
H2A2	4.09	1.07	3.31	1.00	7.92E-06
H3A2	6.99	1.42	6.19	1.20	5.00E-04
H4A2	19.70	3.99	20.19	3.89	5.54E-01

Supplementary Table 3: The concentrations of lipoprotein subclasses in DAS28-classified RA subgroups (Remission, Low, Moderate, High activity) and control, presented with the mean and standard deviation (SD). All lipoprotein species are measured in mg/dL, except particle number (PN), which are measured in nmol/L.

	Control		Remission		Low		Moderate		High	
	**Mean**	**SD**	**Mean**	**SD**	**Mean**	**SD**	**Mean**	**SD**	**Mean**	**SD**
**Total Lipid Content**										
TPTG	97.71	46.34	104.52	45.29	111.80	64.98	126.83	77.38	117.83	53.23
TPCH	196.39	40.20	218.39	39.69	224.63	40.30	212.88	38.18	196.51	37.03
TPA1	153.26	20.52	168.84	27.79	168.50	26.06	162.02	24.01	149.37	25.58
TPA2	32.57	4.22	35.61	5.07	35.58	5.11	34.74	5.77	31.94	5.96
TPAB	82.84	20.16	89.15	17.64	93.20	17.37	90.02	20.11	86.69	17.33
LDHD	1.81	0.52	1.75	0.39	1.83	0.43	1.77	0.58	1.77	0.53
ABA1	0.55	0.14	0.53	0.10	0.56	0.11	0.57	0.15	0.59	0.15
TBPN	1506.24	366.65	1620.96	320.77	1694.68	315.87	1636.76	365.68	1576.19	315.04
**IDL Content**										
IDPN	82.96	34.86	94.33	40.11	96.95	42.42	109.67	52.99	111.11	40.68
IDTG	7.00	6.89	8.34	6.43	9.18	9.18	10.85	10.73	9.07	7.98
IDCH	11.16	5.60	13.00	5.87	13.95	7.43	15.44	8.56	15.03	6.96
IDFC	3.21	1.68	3.76	1.77	3.99	2.20	4.41	2.55	4.21	1.98
IDPL	5.59	2.93	6.86	2.93	6.97	3.35	7.50	4.08	6.32	2.92
IDAB	4.56	1.92	5.19	2.20	5.33	2.33	6.03	2.91	6.11	2.24
**VLDL Content**										
VLPN	130.42	69.70	137.34	65.87	138.22	77.60	164.97	96.54	160.38	62.15
VLTG	59.86	35.25	63.17	33.06	67.67	47.01	80.72	56.99	72.28	39.88
VLCH	17.18	11.02	18.44	9.72	19.78	13.64	22.63	15.37	20.72	11.09
VLFC	7.92	4.42	8.54	4.00	8.95	5.42	10.19	6.38	9.27	4.47
VLPL	17.07	9.36	17.85	8.75	18.21	10.76	21.52	12.75	19.59	9.22
VLAB	7.17	3.83	7.55	3.62	7.60	4.27	9.07	5.31	8.82	3.42
V1TG	22.86	19.44	23.93	19.85	27.42	30.43	34.21	38.32	28.29	27.36
V2TG	9.59	7.33	9.75	6.09	10.62	7.05	12.91	9.14	11.93	6.29
V3TG	9.50	6.73	10.08	5.63	10.57	6.59	12.57	8.13	11.80	5.15
V4TG	8.47	4.18	9.48	4.52	9.14	4.53	11.15	5.39	11.08	3.77
V5TG	2.69	0.83	2.88	1.03	2.82	0.91	3.12	1.00	3.22	0.94
V1CH	4.81	3.91	4.98	3.73	5.83	6.61	6.83	7.91	5.68	5.13
V2CH	2.59	2.03	2.68	1.55	3.04	2.15	3.35	2.62	2.96	1.63
V3CH	3.19	2.44	3.51	2.07	3.74	2.72	4.36	3.20	4.05	2.09
V4CH	4.77	2.85	5.48	2.74	5.57	3.06	6.39	3.29	6.34	2.71
V5CH	1.40	0.73	1.51	0.79	1.41	0.79	1.43	0.83	1.41	0.72
V1FC	1.68	1.60	1.76	1.54	1.90	2.24	2.45	2.63	1.90	1.88
V2FC	1.08	0.92	1.10	0.74	1.23	1.07	1.48	1.39	1.33	0.79
V3FC	1.31	1.08	1.42	0.93	1.52	1.30	1.88	1.56	1.72	0.98
V4FC	1.89	1.28	2.23	1.39	2.24	1.54	2.78	1.74	2.70	1.43
V5FC	0.61	0.43	0.69	0.49	0.70	0.56	0.74	0.81	0.71	0.51
V1PL	3.99	3.19	3.99	3.16	4.50	4.51	5.53	5.36	4.58	4.21
V2PL	2.65	1.88	2.69	1.54	2.86	1.78	3.43	2.32	3.15	1.50
V3PL	3.30	2.23	3.53	1.94	3.52	2.29	4.38	2.75	4.07	1.69
V4PL	4.33	2.11	4.81	2.25	4.69	2.29	5.53	2.63	5.52	1.98
V5PL	1.68	0.82	1.75	0.86	1.68	0.89	1.80	0.85	1.81	0.81

Supplementary Table 3 continued

	Control		Remission		Low		Moderate		High	
	**Mean**	**SD**	**Mean**	**SD**	**Mean**	**SD**	**Mean**	**SD**	**Mean**	**SD**
**LDL Content**										
LDPN	1248.08	326.90	1343.05	274.18	1407.61	284.23	1313.86	325.75	1255.24	268.51
LDTG	19.25	4.61	20.19	5.75	21.32	4.22	22.37	5.70	24.07	5.80
LDCH	105.71	30.92	116.86	24.34	119.72	29.56	108.42	30.77	99.03	25.18
LDFC	31.60	8.14	34.13	6.82	35.89	8.72	32.26	8.30	30.31	7.22
LDPL	61.01	14.92	66.26	12.73	68.00	14.86	62.35	15.17	58.91	12.37
LDAB	68.64	17.98	73.86	15.08	77.42	15.63	72.26	17.92	69.04	14.77
L1TG	6.20	2.02	6.64	2.65	7.05	2.08	7.41	2.67	8.22	2.29
L2TG	2.45	0.73	2.63	0.81	2.67	0.75	2.74	0.78	3.08	0.91
L3TG	2.34	0.67	2.56	0.85	2.66	0.73	2.57	0.74	2.83	0.68
L4TG	2.21	0.97	2.10	0.94	2.15	0.84	2.30	1.03	2.61	1.00
L5TG	2.42	0.84	2.33	0.85	2.50	0.81	2.68	0.94	2.70	0.89
L6TG	3.94	1.08	4.19	1.27	4.52	1.13	4.78	1.51	4.89	1.25
L1CH	23.84	7.19	27.20	8.20	28.06	8.71	25.81	7.60	25.81	7.68
L2CH	15.82	8.30	18.96	6.48	18.98	8.69	15.29	7.33	14.59	6.38
L3CH	15.06	7.17	16.91	5.90	17.67	8.14	14.34	7.05	13.29	5.68
L4CH	13.26	6.65	13.60	4.97	13.65	7.09	11.86	6.96	9.81	5.63
L5CH	14.54	6.46	14.89	5.36	15.11	4.89	14.63	6.30	11.62	4.98
L6CH	22.81	8.32	24.51	7.20	26.46	6.32	26.53	8.42	23.94	5.54
L1FC	7.45	2.09	8.38	2.39	8.82	2.52	8.21	2.22	8.15	2.36
L2FC	5.43	2.36	6.42	2.00	6.54	2.65	5.60	2.13	5.32	2.05
L3FC	5.34	2.05	5.89	1.80	6.19	2.38	5.23	1.98	4.93	1.59
L4FC	4.46	1.63	4.70	1.19	4.78	1.81	4.35	1.73	3.89	1.28
L5FC	4.37	1.54	4.53	1.24	4.69	1.11	4.49	1.52	3.86	1.27
L6FC	5.84	1.89	6.25	1.73	6.86	1.46	6.71	1.82	6.12	1.30
L1PL	13.77	3.58	15.47	4.36	15.88	4.40	14.96	3.84	15.28	3.99
L2PL	9.12	4.09	10.68	3.31	10.66	4.54	8.89	3.70	8.64	3.32
L3PL	8.71	3.52	9.58	2.92	9.97	4.13	8.33	3.59	8.00	2.80
L4PL	7.70	3.38	7.81	2.56	7.87	3.71	7.00	3.61	6.16	2.92
L5PL	8.09	3.17	8.18	2.73	8.29	2.50	8.05	3.17	6.69	2.48
L6PL	12.85	3.99	13.82	3.76	14.74	3.24	14.74	4.04	13.45	2.74
L1AB	12.91	3.44	14.46	4.23	15.06	4.16	14.33	3.73	14.98	3.84
L2AB	8.93	3.93	10.43	3.12	10.46	4.41	8.82	3.58	8.78	3.33
L3AB	9.04	3.69	9.87	3.04	10.37	4.32	8.80	3.81	8.68	2.98
L4AB	8.56	4.09	8.53	3.17	8.71	4.36	7.86	4.39	7.01	3.72
L5AB	10.47	4.33	10.69	3.65	10.99	3.35	10.87	4.31	9.31	3.41
L6AB	18.63	6.80	20.07	5.74	21.82	5.95	22.01	7.47	20.15	4.56
**HDL Content**										
HDTG	9.91	3.09	11.49	4.30	11.23	3.66	12.27	4.18	12.69	3.76
HDCH	59.59	13.78	68.50	16.19	66.85	15.44	63.20	13.76	57.82	12.74
HDFC	13.06	3.52	14.91	4.12	15.48	4.80	14.16	3.76	13.07	3.26
HDPL	80.90	16.03	91.90	21.19	90.84	19.82	87.33	16.93	83.21	15.72
HDA1	152.91	22.36	170.03	29.42	168.39	27.74	161.12	26.03	148.23	27.21
HDA2	32.97	4.12	35.84	4.82	35.87	4.97	35.13	5.69	32.52	5.65
H1TG	3.31	1.72	4.40	2.22	4.17	1.67	4.58	2.03	4.85	1.84
H2TG	1.54	0.63	1.86	0.80	1.77	0.68	2.09	0.78	2.27	0.76
H3TG	1.93	0.73	2.11	0.82	2.10	0.82	2.43	0.84	2.55	0.78
H4TG	3.36	1.21	3.37	1.18	3.40	1.29	3.52	1.27	3.37	1.04

Supplementary Table 3 continued

	Control		Remission		Low		Moderate		High	
	**Mean**	**SD**	**Mean**	**SD**	**Mean**	**SD**	**Mean**	**SD**	**Mean**	**SD**
H1CH	18.65	9.76	23.75	10.40	23.40	10.19	22.43	8.79	21.30	6.66
H2CH	8.25	2.77	10.09	3.08	9.50	3.03	9.45	2.70	8.89	2.29
H3CH	10.53	1.89	11.75	2.38	11.66	2.47	11.46	2.18	10.74	2.43
H4CH	21.49	4.04	22.07	3.74	22.16	3.68	19.92	4.25	17.26	5.53
H1FC	4.42	2.35	5.63	2.49	5.75	2.53	5.25	2.13	4.74	1.57
H2FC	1.95	0.74	2.33	0.79	2.37	0.74	2.33	0.70	2.25	0.56
H3FC	2.21	0.53	2.51	0.65	2.69	0.76	2.52	0.67	2.31	0.65
H4FC	4.12	0.85	4.29	0.88	4.60	1.09	4.15	1.04	3.76	1.24
H1PL	21.96	11.76	28.29	12.75	27.76	12.09	26.93	10.53	26.30	8.15
H2PL	12.68	3.91	15.23	4.46	14.61	4.29	14.81	3.85	14.56	3.31
H3PL	16.73	3.03	18.40	3.72	18.38	3.91	18.31	3.30	17.69	3.59
H4PL	29.05	4.60	29.59	4.34	29.97	4.96	27.60	5.09	25.03	6.40
H1A1	26.91	17.03	35.96	17.43	34.32	16.74	32.82	15.04	30.74	12.52
H2A1	18.86	4.45	21.84	5.68	21.57	5.03	20.99	4.74	20.17	4.16
H3A1	27.34	4.55	29.97	5.42	30.10	6.16	29.78	5.15	28.38	5.89
H4A1	79.84	12.01	81.53	10.42	82.44	11.43	76.38	12.36	68.14	15.83
H1A2	2.49	1.65	3.49	1.86	3.43	1.60	3.41	1.53	3.34	1.20
H2A2	3.31	1.07	4.03	1.23	3.95	1.09	4.23	1.24	4.16	0.93
H3A2	6.19	1.26	6.89	1.31	6.88	1.57	7.21	1.61	6.89	1.52
H4A2	20.19	3.94	20.61	3.27	20.98	3.57	19.42	3.97	17.08	4.76

Supplementary Table 4: Significant lipoproteins and lipoprotein subclasses in DAS28 classified RA patient groups and controls; Multiple comparisons was achieved with one-way ANOVA/Kruskal-Wallis test and *p*-values, following FDR correction (Benjamini, Krieger, Yekutieli), are presented. Significant lipoprotein species ($p < 0.05$) are displayed in red-scaled shading.

	**Control vs. Remission**	**Control vs. Low**	**Control vs. Moderate**	**Control vs. High**	**Remission vs. Low**	**Remission vs. Moderate**	**Remission vs. High**	**Low vs. Moderate**	**Low vs. High**	**Moderate vs. High**
**TPTG**	3.39E-01	5.08E-01	3.78E-02	1.63E-01	6.73E-01	3.39E-01	4.58E-01	1.75E-01	3.39E-01	8.74E-01
**TPCH**	4.27E-02	9.20E-03	6.94E-02	8.32E-01	4.67E-01	4.67E-01	6.94E-02	1.37E-01	2.82E-02	1.37E-01
**HDCH**	2.85E-02	2.85E-02	1.02E-01	6.19E-01	5.75E-01	1.89E-01	2.85E-02	3.01E-01	3.93E-02	1.16E-01
**TPA1**	1.25E-02	1.21E-02	5.17E-02	4.06E-01	6.20E-01	2.27E-01	1.21E-02	2.24E-01	1.21E-02	3.91E-02
**TPA2**	1.92E-02	1.92E-02	3.10E-02	4.49E-01	6.19E-01	3.70E-01	1.92E-02	3.70E-01	1.92E-02	3.10E-02
**TBPN**	3.30E-01	3.07E-02	2.39E-01	3.30E-01	3.30E-01	9.08E-01	9.08E-01	3.30E-01	3.30E-01	9.08E-01
**IDPN**	2.75E-01	2.56E-01	2.77E-02	2.77E-02	6.71E-01	2.75E-01	2.75E-01	3.43E-01	2.75E-01	5.48E-01
**L6PN**	1.98E-01	7.57E-02	1.77E-02	1.98E-01	7.93E-01	7.14E-01	8.73E-01	7.93E-01	7.93E-01	7.93E-01
**IDTG**	2.75E-01	5.68E-01	4.34E-02	4.97E-01	4.97E-01	5.68E-01	6.38E-01	2.33E-01	6.38E-01	4.97E-01
**LDTG**	1.73E-01	2.11E-02	9.10E-03	1.00E-03	1.93E-01	1.47E-01	2.11E-02	5.26E-01	1.18E-01	1.18E-01
**HDTG**	1.04E-01	1.04E-01	2.20E-03	2.70E-03	8.24E-01	2.35E-01	1.63E-01	2.23E-01	1.63E-01	4.60E-01
**IDCH**	3.56E-01	1.14E-01	2.83E-02	1.14E-01	6.87E-01	4.67E-01	5.85E-01	6.87E-01	6.87E-01	9.28E-01
**IDFC**	3.27E-01	1.70E-01	4.07E-02	1.70E-01	7.73E-01	5.94E-01	6.73E-01	6.73E-01	7.73E-01	8.48E-01
**IDPL**	5.07E-02	5.07E-02	1.27E-02	4.22E-01	8.79E-01	7.67E-01	5.95E-01	7.55E-01	5.95E-01	4.22E-01
**HDPL**	3.40E-02	3.40E-02	1.28E-01	5.75E-01	8.11E-01	2.87E-01	1.28E-01	2.87E-01	1.28E-01	3.57E-01
**HDA1**	1.47E-02	1.47E-02	7.80E-02	3.80E-01	5.39E-01	1.99E-01	1.47E-02	2.18E-01	1.47E-02	4.85E-02
**HDA2**	2.73E-02	2.73E-02	3.29E-02	5.11E-01	6.16E-01	4.32E-01	2.73E-02	4.32E-01	2.73E-02	3.39E-02
**IDAB**	2.78E-01	2.54E-01	2.74E-02	2.74E-02	6.74E-01	2.78E-01	2.78E-01	3.44E-01	2.78E-01	5.47E-01
**V4TG**	3.54E-01	3.58E-01	1.61E-02	2.28E-02	7.45E-01	1.22E-01	1.22E-01	1.10E-01	1.10E-01	6.14E-01
**V4CH**	3.03E-01	3.03E-01	2.20E-02	9.21E-02	9.20E-01	3.03E-01	3.03E-01	3.03E-01	3.03E-01	9.20E-01
**V4FC**	3.54E-01	3.54E-01	2.17E-02	1.37E-01	9.33E-01	2.32E-01	3.54E-01	2.32E-01	3.54E-01	9.33E-01
**V4PL**	4.28E-01	4.28E-01	4.45E-02	1.12E-01	8.73E-01	2.72E-01	2.92E-01	2.56E-01	2.72E-01	8.73E-01
**L1TG**	4.52E-01	1.26E-01	9.42E-02	4.70E-03	4.52E-01	3.86E-01	4.53E-02	7.83E-01	9.42E-02	9.42E-02
**L2TG**	1.66E-01	7.22E-02	6.42E-02	1.30E-03	6.50E-01	6.50E-01	6.60E-02	9.10E-01	8.07E-02	7.37E-02
**L6TG**	1.73E-01	3.31E-02	8.00E-03	2.40E-03	3.55E-01	2.31E-01	6.65E-02	5.57E-01	1.73E-01	1.95E-01
**L6CH**	3.07E-01	2.78E-02	1.81E-02	3.37E-01	3.56E-01	3.37E-01	7.53E-01	8.21E-01	3.37E-01	3.37E-01

Supplementary Table 4 continued

	Control vs. Remission	Control vs. Low	Control vs. Moderate	Control vs. High	Remission vs. Low	Remission vs. Moderate	Remission vs. High	Low vs. Moderate	Low vs. High	Moderate vs. High
**L6FC**	2.46E-01	1.24E-02	1.41E-02	3.38E-01	2.46E-01	3.07E-01	6.45E-01	5.00E-01	2.46E-01	2.57E-01
**L6PL**	2.74E-01	2.73E-02	1.69E-02	3.55E-01	3.83E-01	3.60E-01	6.97E-01	8.12E-01	3.55E-01	3.55E-01
**L6AB**	1.98E-01	1.01E-01	1.80E-02	1.98E-01	8.27E-01	7.15E-01	8.70E-01	8.27E-01	8.47E-01	7.97E-01
**H1TG**	1.85E-02	1.55E-02	1.00E-03	1.10E-03	6.19E-01	3.24E-01	2.23E-01	3.24E-01	2.23E-01	3.24E-01
**H2TG**	5.49E-02	6.25E-02	1.00E-04	7.00E-05	6.10E-01	7.87E-02	4.10E-02	5.49E-02	2.53E-02	2.45E-01
**H3TG**	3.31E-01	3.60E-01	5.80E-03	5.80E-03	7.16E-01	9.74E-02	5.99E-02	5.99E-02	5.29E-02	4.24E-01
**H4CH**	3.87E-01	2.97E-01	3.21E-02	6.00E-04	4.14E-01	2.22E-02	5.00E-04	7.40E-03	2.00E-04	2.74E-02
**H1FC**	8.95E-02	3.68E-02	8.95E-02	4.67E-01	6.35E-01	6.35E-01	4.67E-01	4.67E-01	2.80E-01	4.67E-01
**H3FC**	1.11E-01	8.40E-03	9.30E-02	5.57E-01	3.25E-01	9.36E-01	3.25E-01	3.19E-01	9.41E-02	3.25E-01
**H4FC**	5.40E-01	9.01E-02	8.27E-01	2.57E-01	2.57E-01	5.40E-01	1.30E-01	9.01E-02	1.59E-02	2.16E-01
**H1PL**	3.11E-02	3.11E-02	3.11E-02	6.22E-02	7.29E-01	7.29E-01	7.29E-01	7.29E-01	7.29E-01	7.29E-01
**H2PL**	2.69E-02	7.57E-02	2.69E-02	1.41E-01	7.50E-01	7.50E-01	7.50E-01	7.50E-01	8.09E-01	7.50E-01
**H4PL**	6.49E-01	6.49E-01	4.99E-02	3.30E-03	6.49E-01	8.56E-02	5.20E-03	6.23E-02	3.30E-03	6.97E-02
**H4A1**	3.80E-01	3.59E-01	5.83E-02	6.00E-04	4.77E-01	3.24E-02	4.00E-04	1.96E-02	3.00E-04	1.96E-02
**H1A2**	1.88E-02	6.00E-03	6.00E-03	1.88E-02	6.04E-01	6.04E-01	6.04E-01	6.04E-01	6.04E-01	6.04E-01
**H2A2**	7.10E-03	7.10E-03	7.00E-04	7.00E-03	5.63E-01	5.32E-01	5.32E-01	5.19E-01	5.19E-01	5.68E-01
**H3A2**	1.06E-01	1.58E-01	7.50E-03	1.58E-01	7.26E-01	7.26E-01	7.97E-01	4.11E-01	7.97E-01	7.19E-01
**H4A2**	5.41E-01	3.52E-01	2.93E-01	6.10E-03	5.16E-01	2.20E-01	6.10E-03	6.88E-02	1.70E-03	3.61E-02

Supplementary Table 5: Significant lipoproteins and lipoprotein subclasses in DAS28 classified RA patient groups and controls following adjustment for confounding factors, age, gender and CVD. Multiple comparisons was achieved with one-way ANOVA/Kruskal-Wallis test and *p*-values, following FDR correction (Benjamini, Krieger, Yekutieli), are presented. Significant lipoprotein species ($p < 0.05$)are displayed in red-scaled shading.

	**Control vs. Remission**	**Control vs. Low**	**Control vs. Moderate**	**Control vs. High**	**Remission vs. Low**	**Remission vs. Moderate**	**Remission vs. High**	**Low vs. Moderate**	**Low vs. High**	**Moderate vs. High**
**TPTG**	1.26E-01	5.52E-01	1.93E-02	2.21E-01	4.06E-01	5.57E-01	8.28E-01	1.26E-01	5.32E-01	5.52E-01
**TPCH**	1.68E-02	8.00E-04	6.10E-02	4.87E-01	1.78E-01	1.65E-01	2.41E-02	2.11E-02	3.00E-03	9.14E-02
**LDCH**	1.88E-01	2.66E-02	6.94E-01	3.50E-01	3.43E-01	1.88E-01	9.50E-02	2.66E-02	2.66E-02	3.43E-01
**HDCH**	2.00E-03	4.70E-03	1.20E-01	2.18E-01	3.26E-01	2.19E-02	1.90E-03	4.42E-02	2.00E-03	4.00E-02
**TPA1**	3.00E-04	3.00E-04	2.29E-02	8.26E-02	2.86E-01	1.43E-02	4.91E-04	1.43E-02	1.00E-04	3.80E-03
**TPA2**	8.20E-03	8.20E-03	8.38E-02	4.24E-01	6.16E-01	1.25E-01	8.20E-03	1.25E-01	8.20E-03	6.52E-02
**TPAB**	2.24E-01	3.88E-02	2.24E-01	5.32E-01	5.32E-01	6.34E-01	5.32E-01	3.28E-01	2.37E-01	5.45E-01
**TBPN**	5.70E-02	1.52E-02	1.71E-01	5.19E-01	5.96E-01	4.02E-01	2.46E-01	1.83E-01	1.71E-01	5.40E-01
**IDPN**	2.59E-01	2.77E-01	3.61E-02	3.61E-02	6.22E-01	3.41E-01	2.77E-01	2.59E-01	2.59E-01	5.63E-01
**L1PN**	1.29E-01	1.74E-02	1.93E-01	1.29E-01	5.27E-01	5.43E-01	8.06E-01	1.63E-01	5.72E-01	5.27E-01
**L6PN**	1.30E-01	7.49E-02	2.34E-02	4.58E-01	7.74E-01	6.76E-01	6.76E-01	7.73E-01	6.40E-01	4.58E-01
**VLTG**	2.69E-01	6.55E-01	2.14E-02	5.94E-01	4.39E-01	4.77E-01	6.22E-01	6.52E-02	6.55E-01	2.69E-01
**IDTG**	8.08E-02	4.03E-01	4.00E-02	4.03E-01	3.61E-01	8.96E-01	4.03E-01	2.63E-01	8.05E-01	4.03E-01
**LDTG**	1.94E-01	6.31E-02	2.82E-02	1.87E-02	5.97E-01	5.39E-01	2.40E-01	7.41E-01	3.45E-01	3.45E-01
**HDTG**	4.70E-03	3.62E-02	9.00E-04	5.30E-03	3.39E-01	7.07E-01	7.07E-01	3.34E-01	3.39E-01	7.07E-01
**LDFC**	2.44E-01	9.80E-03	6.81E-01	4.60E-01	1.97E-01	2.12E-01	1.97E-01	9.80E-03	9.80E-03	4.60E-01
**HDFC**	9.20E-03	1.40E-03	1.21E-01	3.18E-01	2.83E-01	7.72E-02	9.20E-03	9.40E-03	1.40E-03	8.17E-02
**LDPL**	1.72E-01	2.52E-02	6.95E-01	4.64E-01	3.70E-01	1.72E-01	1.38E-01	2.52E-02	2.52E-02	4.64E-01
**IDPL**	3.35E-02	3.35E-02	1.70E-02	3.38E-01	6.47E-01	7.24E-01	3.38E-01	6.47E-01	3.90E-01	3.38E-01
**HDPL**	1.10E-03	1.10E-03	4.36E-02	6.02E-01	6.02E-01	4.01E-02	4.70E-03	4.01E-02	4.70E-03	1.01E-01
**HDA1**	7.00E-04	1.00E-03	5.31E-02	1.15E-01	3.12E-01	1.80E-02	2.00E-04	2.81E-02	2.00E-04	9.50E-03
**HDA2**	9.80E-03	9.00E-03	6.58E-02	4.58E-01	5.47E-01	1.63E-01	9.80E-03	1.12E-01	9.00E-03	6.31E-02
**IDAB**	2.61E-01	2.76E-01	3.60E-02	3.60E-02	6.22E-01	3.42E-01	2.76E-01	2.61E-01	2.61E-01	5.61E-01
**V3TG**	2.52E-01	4.63E-01	3.14E-02	1.92E-01	6.32E-01	4.92E-01	6.32E-01	2.52E-01	4.66E-01	7.80E-01
**V4TG**	2.77E-01	5.00E-01	2.26E-02	1.02E-01	5.00E-01	3.60E-01	5.00E-01	1.02E-01	2.77E-01	8.14E-01
**V5TG**	4.93E-01	5.78E-01	4.00E-02	4.48E-01	6.66E-01	4.48E-01	6.77E-01	2.08E-01	5.78E-01	5.78E-01
**V4CH**	2.52E-01	2.52E-01	1.17E-02	1.22E-01	7.70E-01	4.50E-01	6.25E-01	2.52E-01	4.72E-01	7.70E-01
**V4FC**	3.75E-01	4.15E-01	2.81E-02	2.25E-01	7.73E-01	3.75E-01	5.11E-01	2.99E-01	4.15E-01	7.73E-01
**V3PL**	3.23E-01	5.09E-01	4.35E-02	1.51E-01	5.09E-01	4.31E-01	5.09E-01	1.51E-01	3.23E-01	8.20E-01
**L1TG**	2.02E-01	2.02E-01	3.25E-02	3.25E-02	8.20E-01	4.83E-01	3.19E-01	4.83E-01	3.19E-01	4.83E-01
**L2TG**	1.15E-01	1.15E-01	1.15E-01	8.10E-03	7.62E-01	7.62E-01	1.85E-01	6.87E-01	2.17E-01	1.15E-01
**L6TG**	6.44E-02	4.70E-02	2.20E-03	9.50E-03	7.69E-01	3.61E-01	3.61E-01	3.61E-01	3.61E-01	7.12E-01
**L1CH**	1.62E-01	1.89E-02	4.04E-01	4.04E-01	4.04E-01	4.04E-01	5.45E-01	6.85E-02	2.87E-01	6.75E-01
**L2CH**	2.66E-01	8.34E-02	6.44E-01	7.74E-01	5.93E-01	1.03E-01	2.64E-01	2.32E-02	8.34E-02	8.48E-01
**L3CH**	4.79E-01	1.28E-01	4.28E-01	4.79E-01	3.79E-01	2.10E-01	3.14E-01	1.53E-02	6.23E-02	9.07E-01
**L6CH**	1.93E-01	3.19E-02	2.29E-02	4.44E-01	4.44E-01	4.44E-01	4.44E-01	8.29E-01	2.18E-01	2.10E-01
**L1FC**	1.87E-01	6.10E-03	2.93E-01	2.93E-01	2.93E-01	4.81E-01	6.99E-01	6.84E-02	2.31E-01	7.60E-01
**L3FC**	3.66E-01	5.49E-02	4.26E-01	4.26E-01	3.40E-01	1.61E-01	1.68E-01	1.09E-02	2.59E-02	6.54E-01
**L6FC**	1.76E-01	1.46E-02	2.02E-02	4.17E-01	2.99E-01	4.17E-01	4.17E-01	4.17E-01	1.40E-01	2.01E-01
**L1PL**	9.59E-02	6.60E-03	3.39E-01	2.09E-01	3.61E-01	3.39E-01	7.81E-01	5.98E-02	3.39E-01	4.29E-01
**L2PL**	2.57E-01	1.08E-01	6.79E-01	8.06E-01	6.43E-01	1.08E-01	2.57E-01	2.79E-02	1.08E-01	8.06E-01
**L3PL**	5.34E-01	1.57E-01	4.02E-01	5.34E-01	4.02E-01	2.00E-01	4.02E-01	1.72E-02	1.10E-01	8.43E-01
**L6PL**	1.44E-01	3.55E-02	1.62E-02	4.87E-01	5.51E-01	4.87E-01	4.87E-01	7.40E-01	2.60E-01	2.04E-01
**L1AB**	1.29E-01	1.73E-02	1.92E-01	1.29E-01	5.28E-01	5.45E-01	8.05E-01	1.63E-01	5.73E-01	5.28E-01
**L3AB**	6.31E-01	1.16E-01	6.31E-01	8.83E-01	3.61E-01	3.61E-01	6.31E-01	2.92E-02	2.33E-01	6.31E-01
**L6AB**	1.31E-01	9.62E-02	2.36E-02	4.61E-01	8.15E-01	6.75E-01	6.75E-01	7.28E-01	6.75E-01	4.61E-01
**H1TG**	9.00E-04	4.60E-03	9.00E-04	4.80E-03	5.42E-01	5.42E-01	5.42E-01	5.42E-01	5.42E-01	5.42E-01
**H2TG**	6.70E-03	1.18E-01	1.00E-04	9.00E-04	1.48E-01	3.36E-01	2.76E-01	2.82E-02	3.37E-02	4.23E-01
**H3TG**	9.01E-02	2.62E-01	3.30E-03	1.15E-02	4.06E-01	2.62E-01	2.62E-01	9.01E-02	9.01E-02	6.26E-01
**H2CH**	4.60E-03	3.48E-02	4.00E-02	4.65E-01	3.57E-01	1.58E-01	4.00E-02	4.65E-01	1.58E-01	2.65E-01
**H3CH**	1.06E-02	1.06E-02	3.97E-02	6.04E-01	6.04E-01	2.03E-01	2.63E-02	2.08E-01	2.63E-02	1.00E-01
**H4CH**	3.81E-01	2.73E-01	2.53E-02	5.00E-04	3.96E-01	1.68E-02	3.00E-04	4.50E-03	1.00E-04	2.53E-02
**H1FC**	3.51E-02	1.81E-02	1.36E-01	5.51E-01	6.64E-01	2.46E-01	1.36E-01	1.36E-01	1.21E-01	4.42E-01
**H2FC**	2.11E-02	4.80E-03	2.11E-02	1.78E-01	4.96E-01	5.41E-01	4.96E-01	3.46E-01	3.18E-01	5.36E-01
**H4FC**	2.24E-01	2.49E-02	6.12E-01	8.98E-02	1.83E-01	2.14E-01	2.49E-02	2.49E-02	1.70E-03	8.98E-02
**H1PL**	8.80E-03	1.79E-02	3.35E-02	1.57E-01	4.74E-01	3.19E-01	3.28E-01	4.72E-01	4.72E-01	6.46E-01
**H2PL**	2.30E-03	8.90E-03	8.90E-03	1.81E-01	3.71E-01	3.04E-01	1.81E-01	6.02E-01	3.71E-01	3.89E-01
**H3PL**	2.15E-02	2.15E-02	4.29E-02	4.43E-01	7.53E-01	4.43E-01	1.89E-01	4.43E-01	1.89E-01	3.67E-01
**H4PL**	3.91E-01	2.91E-01	5.81E-02	8.00E-04	4.01E-01	3.18E-02	6.00E-04	1.34E-02	2.00E-04	2.05E-02
**H1A1**	1.49E-02	3.57E-02	9.31E-02	3.33E-01	5.03E-01	2.62E-01	2.41E-01	4.50E-01	3.33E-01	5.10E-01
**H2A1**	2.40E-03	3.60E-03	2.47E-02	3.52E-01	3.98E-01	1.23E-01	2.58E-02	2.11E-01	4.87E-02	1.68E-01
**H3A1**	1.65E-02	1.65E-02	4.18E-02	6.78E-01	8.38E-01	3.87E-01	5.76E-02	3.87E-01	5.58E-02	1.58E-01
**H4A1**	2.68E-01	2.24E-01	2.94E-02	2.00E-04	3.57E-01	1.19E-02	1.00E-04	6.10E-03	3.17E-05	1.19E-02
**H1A2**	9.40E-03	8.20E-03	1.21E-02	6.46E-02	7.19E-01	5.30E-01	5.30E-01	5.30E-01	5.30E-01	7.19E-01
**H2A2**	1.50E-03	1.50E-03	4.00E-04	2.50E-03	6.25E-01	6.25E-01	6.25E-01	6.25E-01	6.25E-01	6.25E-01
**H3A2**	2.13E-02	5.55E-02	1.02E-02	1.31E-01	5.72E-01	8.00E-01	5.72E-01	5.72E-01	8.00E-01	5.72E-01
**H4A2**	5.84E-01	3.73E-01	2.65E-01	5.50E-03	4.96E-01	2.30E-01	5.50E-03	6.63E-02	1.70E-03	3.79E-02

Supplementary Table 6: Statistical significant lipoprotein and lipoprotein subclasses (*p<0.05*) in DAS28 classified RA patient groups and controls; *p*-values for each comparison pair, mean and standard deviation of each group and the calculated power (0.05) are presented. Comparisons with power values below 0.80 are considered not significant and are underlined. Each group sample size is written in parenthesis.

**Control vs. High**		**Control (46)**		**High (23)**		
	p-value	**Mean**	**Std Dev**	**Mean**	**Std Dev**	**Power (0.05)**
**LDTG**	1.00E-03	19.25	4.606	24.07	5.796	0.957
**HDTG**	2.70E-03	9.91	3.085	12.693	3.756	0.896
**IDPN**	2.77E-02	82.958	34.858	111.105	40.685	0.835
**L1TG**	4.70E-03	6.196	2.023	8.225	2.295	0.958
**L2TG**	1.30E-03	2.451	0.729	3.08	0.908	0.862
**L6TG**	2.40E-03	3.942	1.08	4.892	1.249	0.893
**H1TG**	1.10E-03	3.307	1.724	4.849	1.837	0.920
**H2TG**	7.00E-05	1.541	0.632	2.275	0.762	0.986
**H3TG**	5.80E-03	1.933	0.727	2.554	0.782	0.893
**H4CH**	6.00E-04	21.485	4.04	17.258	5.526	0.943
**H4PL**	3.30E-03	29.047	4.595	25.026	6.402	0.835
**H4A1**	6.00E-04	79.842	12.009	68.139	15.83	0.919
**H1A2**	1.88E-02	2.491	1.653	3.338	1.198	<u>0.575</u>
**H2A2**	7.00E-03	3.313	1.066	4.16	0.931	0.889
**H4A2**	6.10E-03	20.191	3.939	17.083	4.759	0.806

**Remission vs. High**		**Remission (33)**		**High (23)**		
	p-value	**Mean**	**Std Dev**	**Mean**	**Std Dev**	**Power (0.05)**
**HDCH**	2.85E-02	68.505	16.195	57.821	12.737	<u>0.737</u>
**TPA1**	1.21E-02	168.845	27.79	149.37	25.583	<u>0.744</u>
**TPA2**	1.92E-02	35.607	5.068	31.94	5.965	<u>0.681</u>
**LDTG**	2.11E-02	20.19	5.745	24.07	5.796	<u>0.682</u>
**HDA1**	1.47E-02	170.025	29.418	148.229	27.214	<u>0.789</u>
**HDA2**	2.73E-02	35.836	4.82	2.52	5.647	<u>0.640</u>
**L1TG**	4.53E-02	6.635	2.646	8.225	2.295	<u>0.629</u>
**H2TG**	4.10E-02	1.862	0.801	2.275	0.762	<u>0.476</u>
**H4CH**	5.00E-04	22.072	3.738	17.258	5.526	0.969
**H4PL**	5.20E-03	29.592	4.344	25.026	6.402	0.878
**H4A1**	4.00E-04	81.528	10.423	68.139	15.83	0.963
**H4A2**	6.10E-03	20.608	3.272	17.083	4.759	0.898

Supplementary Table 6 continued

**Low vs. High**		**Low (40)**		**High (23)**		
	p-value	**Mean**	**Std Dev**	**Mean**	**Std Dev**	**Power (0.05)**
**TPCH**	2.82E-02	224.625	40.301	196.507	37.027	0.771
**HDCH**	3.93E-02	66.85	15.437	57.821	12.737	0.647
**TPA1**	1.21E-02	168.499	26.056	149.37	25.583	0.794
**TPA2**	1.92E-02	35.58	5.111	31.94	5.965	0.712
**HDA1**	1.47E-02	168.395	27.74	148.229	27.214	0.786
**HDA2**	2.73E-02	35.87	4.966	32.52	5.647	0.675
**H2TG**	2.53E-02	1.77	0.683	2.275	0.762	0.759
**H4CH**	2.00E-04	22.16	3.679	17.258	5.526	0.986
**H4FC**	1.59E-02	4.603	1.094	3.76	1.237	0.789
**H4PL**	3.30E-03	29.973	4.96	25.026	6.402	0.920
**H4A1**	3.00E-04	82.438	11.435	68.139	15.83	0.983
**H4A2**	1.70E-03	20.983	3.574	17.083	4.759	0.952
**Moderate vs. High**		**Moderate (63)**		**High (23)**		
	p-value	**Mean**	**Std Dev**	**Mean**	**Std Dev**	**Power (0.05)**
**TPA1**	3.91E-02	161.829	23.997	149.37	25.583	0.544
**TPA2**	3.10E-02	34.561	5.59	31.94	5.965	0.464
**HDA1**	4.85E-02	161.135	26.057	148.229	27.214	0.511
**HDA2**	3.39E-02	34.971	5.55	32.52	5.647	0.430
**H4CH**	2.74E-02	19.955	4.337	17.258	5.526	0.648
**H4A1**	1.96E-02	76.433	12.636	68.139	15.83	0.700
**H4A2**	3.61E-02	19.362	3.976	17.083	4.759	0.597

Supplementary Table 7: Predictive performance of selected lipoproteins in discriminating between rheumatoid arthritis patients in remission and those with high disease activity. The area under the receiver operating characteristic curve (AUC) are presented for each lipoprotein variable, reflecting its discriminative ability. *p*-values indicate the statistical significance of the observed AUCs compared to the null hypothesis of no discriminatory power (AUC = 0.5).

**Lipoprotein**	**AUC**	***p*-value**
H4A1	0.80	3.46E-04
H4CH	0.78	2.74E-04
H4A2	0.75	1.78E-03
H4PL	0.73	2.42E-03
LDCH	0.71	1.03E-02
HDA1	0.71	6.86E-03
TPA1	0.70	1.02E-02

Supplementary Table 8: The *p*-values of the partial correlation of IVDr lipoprotein subclasses with clinical markers DAS28, CRP and VAS, following correction for age, gender and CVD. Significant lipoprotein species ($p < 0.05$) are displayed in red-scaled shading.

	DAS28		CRP		VAS	
	r-corr	*p*-value	r-corr	*p*-value	r-corr	*p*-value
**Total Lipid Content**						
**TPTG**	0.07	4.17E-01	-0.04	6.56E-01	0.05	5.43E-01
**TPCH**	-0.24	2.39E-03	-0.20	1.18E-02	-0.15	6.18E-02
**TPA1**	-0.39	6.62E-07	-0.34	1.90E-05	-0.20	1.19E-02
**TPA2**	-0.25	1.37E-03	-0.24	2.35E-03	-0.19	1.51E-02
**TPAB**	-0.06	4.72E-01	-0.07	3.76E-01	-0.04	6.18E-01
**LDHD**	0.08	3.34E-01	0.05	5.40E-01	0.00	9.55E-01
**ABA1**	0.21	9.58E-03	0.13	1.01E-01	0.09	2.38E-01
**TBPN**	-0.06	4.72E-01	-0.07	3.76E-01	-0.04	6.18E-01
**IDL Content**						
**IDPN**	0.14	7.74E-02	0.01	9.03E-01	0.15	5.57E-02
**IDTG**	0.02	8.52E-01	-0.07	3.90E-01	-0.01	9.21E-01
**IDCH**	0.11	1.80E-01	-0.01	9.46E-01	0.11	1.53E-01
**IDFC**	0.08	2.92E-01	-0.02	7.90E-01	0.09	2.68E-01
**IDPL**	-0.05	5.74E-01	-0.11	1.67E-01	-0.05	5.32E-01
**IDAB**	0.14	7.74E-02	0.01	9.03E-01	0.15	5.57E-02
**VLDL Content**						
**VLPN**	0.10	2.01E-01	0.01	8.92E-01	0.05	5.19E-01
**VLTG**	0.07	3.68E-01	-0.01	8.71E-01	0.03	7.19E-01
**VLCH**	0.07	4.14E-01	-0.03	6.96E-01	0.03	6.67E-01
**VLFC**	0.05	5.73E-01	-0.04	6.47E-01	0.02	7.79E-01
**VLPL**	0.06	4.21E-01	-0.01	9.03E-01	0.02	8.24E-01
**VLAB**	0.10	2.01E-01	0.01	8.92E-01	0.05	5.19E-01
**V1TG**	0.06	4.54E-01	-0.02	7.62E-01	0.05	5.77E-01
**V2TG**	0.07	3.66E-01	0.00	9.81E-01	-0.05	5.07E-01
**V3TG**	0.08	3.16E-01	0.02	8.08E-01	-0.02	7.75E-01
**V4TG**	0.12	1.35E-01	0.07	4.14E-01	0.06	4.70E-01
**V5TG**	0.07	3.77E-01	0.04	6.50E-01	0.09	2.78E-01
**V1CH**	0.06	4.82E-01	-0.05	5.58E-01	0.06	4.31E-01
**V2CH**	0.05	5.76E-01	-0.06	4.58E-01	-0.02	8.11E-01
**V3CH**	0.08	3.08E-01	0.01	9.36E-01	0.01	8.77E-01
**V4CH**	0.09	2.45E-01	0.02	7.85E-01	0.06	4.87E-01
**V5CH**	-0.09	2.63E-01	-0.05	5.15E-01	-0.07	3.78E-01
**V1FC**	0.04	5.88E-01	-0.05	5.21E-01	0.02	7.67E-01
**V2FC**	0.09	2.77E-01	-0.02	8.52E-01	0.03	7.26E-01
**V3FC**	0.09	2.44E-01	0.01	9.10E-01	0.04	6.53E-01
**V4FC**	0.11	1.81E-01	0.01	8.59E-01	0.08	3.13E-01
**V5FC**	0.01	8.72E-01	-0.06	4.70E-01	0.09	2.86E-01
**V1PL**	0.06	4.67E-01	-0.03	7.15E-01	0.02	8.01E-01
**V2PL**	0.08	3.34E-01	0.00	9.66E-01	-0.05	5.73E-01

Supplementary Table 8 continued

	DAS28		CRP		VAS	
	r-corr	*p*-value	r-corr	*p*-value	r-corr	*p*-value
**V3PL**	0.10	1.95E-01	0.03	7.16E-01	0.02	8.46E-01
**V4PL**	0.11	1.90E-01	0.03	6.84E-01	0.06	4.73E-01
**V5PL**	-0.02	8.25E-01	0.00	9.86E-01	-0.02	8.35E-01
**LDL Content**						
**LDPN**	-0.11	1.79E-01	-0.07	3.64E-01	-0.09	2.85E-01
**LDTG**	0.21	8.09E-03	0.06	4.28E-01	0.18	2.38E-02
**LDCH**	-0.22	6.48E-03	-0.13	1.00E-01	-0.16	4.85E-02
**LDFC**	-0.21	8.34E-03	-0.12	1.33E-01	-0.15	6.04E-02
**LDPL**	-0.20	1.30E-02	-0.12	1.50E-01	-0.15	6.43E-02
**LDAB**	-0.11	1.80E-01	-0.07	3.64E-01	-0.09	2.85E-01
**L1TG**	0.15	6.45E-02	0.02	8.52E-01	0.17	3.54E-02
**L2TG**	0.14	7.57E-02	0.11	1.91E-01	0.16	4.60E-02
**L3TG**	0.05	5.47E-01	0.04	6.08E-01	0.08	3.28E-01
**L4TG**	0.18	2.73E-02	0.07	3.60E-01	0.12	1.52E-01
**L5TG**	0.18	2.59E-02	0.03	7.19E-01	0.11	1.59E-01
**L6TG**	0.18	2.80E-02	0.04	5.92E-01	0.08	3.24E-01
**L1CH**	-0.15	5.60E-02	-0.10	2.34E-01	-0.06	4.81E-01
**L2CH**	-0.22	6.07E-03	-0.10	2.28E-01	-0.14	8.75E-02
**L3CH**	-0.20	1.13E-02	-0.06	4.91E-01	-0.15	5.58E-02
**L4CH**	-0.17	3.13E-02	-0.10	1.99E-01	-0.14	8.67E-02
**L5CH**	-0.11	1.85E-01	-0.12	1.21E-01	-0.10	2.04E-01
**L6CH**	0.02	8.29E-01	-0.04	5.96E-01	-0.01	8.97E-01
**L1FC**	-0.13	9.30E-02	-0.10	2.30E-01	-0.04	6.62E-01
**L2FC**	-0.20	1.33E-02	-0.11	1.74E-01	-0.10	2.07E-01
**L3FC**	-0.22	5.70E-03	-0.08	2.97E-01	-0.15	6.43E-02
**L4FC**	-0.18	2.62E-02	-0.11	1.78E-01	-0.14	9.26E-02
**L5FC**	-0.10	2.01E-01	-0.12	1.29E-01	-0.10	2.33E-01
**L6FC**	-0.02	8.13E-01	-0.04	6.00E-01	-0.06	4.58E-01
**L1PL**	-0.12	1.45E-01	-0.08	3.25E-01	-0.03	7.41E-01
**L2PL**	-0.21	9.57E-03	-0.09	2.71E-01	-0.13	1.20E-01
**L3PL**	-0.19	1.93E-02	-0.05	5.50E-01	-0.14	7.89E-02
**L4PL**	-0.15	5.94E-02	-0.09	2.75E-01	-0.13	1.11E-01
**L5PL**	-0.09	2.50E-01	-0.12	1.38E-01	-0.10	2.26E-01
**L6PL**	0.00	9.89E-01	-0.06	4.84E-01	-0.02	8.22E-01
**L1AB**	-0.06	4.82E-01	-0.03	6.86E-01	0.02	8.47E-01
**L2AB**	-0.18	2.72E-02	-0.07	3.92E-01	-0.11	1.87E-01
**L3AB**	-0.15	6.82E-02	-0.02	8.36E-01	-0.12	1.46E-01
**L4AB**	-0.11	1.64E-01	-0.08	3.44E-01	-0.10	2.02E-01
**L5AB**	-0.04	6.19E-01	-0.09	2.59E-01	-0.06	4.75E-01
**L6AB**	0.04	5.85E-01	-0.04	6.25E-01	0.02	8.01E-01

Supplementary Table 8 continued

	DAS28		CRP		VAS	
	**r-corr**	***p*-value**	**r-corr**	***p*-value**	**r-corr**	***p*-value**
**HDL Content**						
**HDTG**	0.01	9.24E-01	-0.03	6.70E-01	0.04	5.91E-01
**HDCH**	-0.34	1.34E-05	-0.25	1.43E-03	-0.19	1.59E-02
**HDFC**	-0.31	7.36E-05	-0.23	3.62E-03	-0.16	4.29E-02
**HDPL**	-0.30	1.10E-04	-0.22	5.22E-03	-0.18	2.61E-02
**HDA1**	-0.40	3.09E-07	-0.34	1.07E-05	-0.21	7.51E-03
**HDA2**	-0.23	3.24E-03	-0.23	3.54E-03	-0.18	2.88E-02
**H1TG**	-0.03	7.54E-01	-0.03	7.10E-01	0.06	4.26E-01
**H2TG**	0.11	1.83E-01	0.06	4.75E-01	0.12	1.38E-01
**H3TG**	0.13	9.84E-02	0.03	6.86E-01	0.11	1.59E-01
**H4TG**	-0.03	7.12E-01	-0.10	1.94E-01	-0.03	6.66E-01
**H1CH**	-0.18	2.35E-02	-0.10	2.16E-01	-0.06	4.45E-01
**H2CH**	-0.22	6.01E-03	-0.13	1.16E-01	-0.10	2.16E-01
**H3CH**	-0.22	5.27E-03	-0.17	3.05E-02	-0.14	8.74E-02
**H4CH**	-0.38	9.73E-07	-0.33	2.69E-05	-0.31	1.07E-04
**H1FC**	-0.23	3.80E-03	-0.16	5.13E-02	-0.11	1.60E-01
**H2FC**	-0.12	1.26E-01	-0.07	3.66E-01	-0.07	3.67E-01
**H3FC**	-0.22	6.62E-03	-0.16	5.07E-02	-0.18	2.43E-02
**H4FC**	-0.26	1.19E-03	-0.19	1.94E-02	-0.26	1.21E-03
**H1PL**	-0.17	3.84E-02	-0.09	2.84E-01	-0.05	4.96E-01
**H2PL**	-0.15	5.93E-02	-0.07	3.68E-01	-0.05	5.02E-01
**H3PL**	-0.16	4.59E-02	-0.13	1.05E-01	-0.10	1.98E-01
**H4PL**	-0.34	1.49E-05	-0.31	1.02E-04	-0.29	2.30E-04
**H1A1**	-0.21	9.31E-03	-0.14	7.16E-02	-0.07	3.89E-01
**H2A1**	-0.23	4.44E-03	-0.19	1.61E-02	-0.08	3.01E-01
**H3A1**	-0.20	1.43E-02	-0.18	2.27E-02	-0.08	2.97E-01
**H4A1**	-0.39	4.58E-07	-0.38	1.20E-06	-0.29	3.09E-04
**H1A2**	-0.11	1.74E-01	-0.06	4.39E-01	-0.03	6.94E-01
**H2A2**	0.03	7.22E-01	0.03	7.37E-01	0.06	4.52E-01
**H3A2**	0.00	9.66E-01	-0.03	6.76E-01	-0.01	9.37E-01
**H4A2**	-0.29	2.17E-04	-0.28	3.27E-04	-0.26	1.27E-03

Supplementary Table 9: p-values for pairwise comparisons of glycoprotein and SPC signal levels across rheumatoid arthritis (RA) disease activity groups and controls. Statistical significance between groups was assessed using one-way ANOVA/Kruskal–Wallis tests, followed by correction for multiple comparisons. The table presents adjusted p-values for all pairwise comparisons among controls and RA subgroups (remission, low, moderate, and high disease activity). Statistically significant differences (p <0.05) are underlined.

	Remission vs. Control	Low vs. Control	Moderate vs. Control	High vs. Control	Remission vs. Low	Remission vs. Moderate	Remission vs. High	Low vs. Moderate	Low vs. High	Moderate vs. High
**GlycA**	1.31E-01	4.16E-02	1.35E-06	1.82E-05	2.64E-01	4.00E-04	1.40E-03	1.60E-03	3.80E-03	2.96E-01
**GlycB**	2.40E-01	9.40E-02	1.07E-04	3.00E-04	2.81E-01	1.28E-02	3.40E-03	4.75E-02	1.07E-02	1.41E-01
**GlycA sum**	9.67E-02	3.92E-02	4.18E-05	3.00E-04	3.80E-01	1.35E-02	1.93E-02	2.15E-02	2.82E-02	3.80E-01
**GlycB sum**	1.92E-01	1.01E-01	5.93E-06	1.64E-05	2.87E-01	8.00E-04	8.00E-04	1.50E-03	1.40E-03	1.92E-01
**GlycA + B**	1.27E-01	3.53E-02	3.18E-07	6.50E-06	2.25E-01	8.00E-04	9.00E-04	2.90E-03	2.90E-03	2.25E-01
**SPC sum**	7.84E-02	6.89E-02	7.84E-02	6.03E-01	8.49E-01	7.81E-01	5.79E-01	6.10E-01	4.70E-01	6.03E-01
**SPC sum/ GlycA**	7.68E-01	7.68E-01	7.68E-01	9.58E-01	8.96E-01	7.69E-01	7.68E-01	8.96E-01	7.68E-01	7.69E-01

Supplementary Table 10: Differences and predictive performance of glycoprotein and SPC markers in DMARD-naïve rheumatoid arthritis (RA) patients compared to controls. The table presents *p*-values from statistical comparisons, evaluated with unpaired two-tailed t-test/Mann-Whitney test, and the corresponding area under the curve (AUC) values reflecting each marker's ability to distinguish DMARD-naïve RA patients from healthy controls. *p*-values <0.05 suggest statistical significance, while AUC values closer to 1 indicate stronger discriminatory power.

	***p*-value**	**AUC**
**GlycA**	2.74E-05	0.88
**GlycB**	2.42E-06	0.88
**GlycA+B total**	3.79E-06	0.90
**SPC sum**	6.09E-01	0.58
**SPC total/GlycA**	8.51E-02	0.67

Supplementary Table 11: Partial correlation of glycoproteins and SPC signals with classical clinical parameters, DAS28, CRP and VAS, following correction for age, gender and CVD presence. The statistical significance of correlations was evaluated with the *p*-value. Significant *p*-values ($p < 0.05$) are underlined.

	**DAS28**		**CRP**		**VAS**	
	r-corr	*p*-value	r-corr	*p*-value	r-corr	*p*-value
**GlycA**	0.31	8.23E-05	0.21	8.40E-03	0.21	9.35E-03
**GlycA sum**	0.19	1.81E-02	0.09	2.67E-01	0.15	6.44E-02
**GlycB**	0.29	2.53E-04	0.18	2.26E-02	0.24	2.66E-03
**GlycB Sum**	0.28	3.59E-04	0.16	5.15E-02	0.25	1.97E-03
**SPC sum**	-0.27	6.34E-04	-0.21	1.08E-02	-0.16	4.92E-02
**SPC total/ GlycA**	-0.05	5.67E-01	-0.07	3.78E-01	-0.04	5.83E-01

Supplementary Table 12: The *p*-values of the partial correlation of Glycoproteins and SPC signals with IVDr lipoprotein subclasses following correction for age, gender and CVD presence. Statistically significant correlation based on the *p*-value are identified in red-scaled shading.

	GlycA	GlycA sum	GlycB	GlycB Sum	SPC sum	SPC total/GlycA
**Total Lipid Content**						
TPTG	2.1E-26	1.3E-47	1.7E-12	6.4E-18	7.3E-01	3.2E-01
TPCH	1.0E-02	2.0E-05	6.9E-03	3.3E-03	2.6E-27	3.1E-01
TPA1	1.1E-01	6.2E-01	4.8E-01	6.6E-01	2.1E-39	8.1E-01
TPA2	3.4E-02	2.7E-04	8.4E-03	7.1E-04	2.7E-20	9.1E-01
TPAB	4.0E-04	1.7E-07	2.5E-03	2.2E-03	2.5E-07	1.3E-01
LDHD	1.9E-01	9.0E-02	3.8E-01	5.6E-01	6.0E-01	3.5E-01
ABA1	2.9E-05	1.1E-06	2.4E-03	3.1E-03	7.9E-02	1.3E-01
TBPN	4.0E-04	1.7E-07	2.5E-03	2.2E-03	2.5E-07	1.3E-01
**IDL Content**						
IDPN	8.2E-20	1.9E-33	4.1E-20	4.8E-24	3.0E-02	1.3E-01
IDTG	2.3E-22	1.6E-39	3.7E-09	3.7E-13	3.7E-01	3.5E-01
IDCH	1.3E-24	2.7E-44	3.7E-23	1.7E-27	2.1E-02	1.6E-01
IDFC	5.5E-25	2.1E-46	4.3E-21	2.5E-25	1.9E-02	1.6E-01
IDPL	2.6E-23	5.5E-44	5.6E-14	3.0E-17	7.7E-05	1.1E-01
IDAB	8.1E-20	1.9E-33	4.1E-20	4.7E-24	3.0E-02	1.3E-01
**VLDL Content**						
VLPN	2.5E-24	1.2E-38	2.5E-10	2.2E-13	6.1E-01	1.6E-01
VLTG	1.1E-27	1.7E-47	5.7E-13	2.8E-18	7.5E-01	2.7E-01
VLCH	5.7E-28	2.0E-49	1.0E-12	4.4E-17	7.0E-01	2.6E-01
VLFC	3.5E-25	1.4E-44	3.6E-11	3.5E-15	8.2E-01	2.6E-01
VLPL	1.6E-24	6.3E-41	8.7E-11	1.4E-14	7.7E-01	2.1E-01
VLAB	2.5E-24	1.2E-38	2.5E-10	2.2E-13	6.1E-01	1.6E-01
V1TG	4.8E-23	1.2E-38	3.3E-12	9.9E-19	4.1E-01	5.6E-01
V2TG	2.0E-20	2.5E-26	2.6E-06	1.0E-07	9.7E-01	9.2E-02
V3TG	6.5E-22	9.4E-30	1.8E-08	5.5E-10	9.2E-01	5.6E-02
V4TG	9.5E-18	1.2E-23	4.7E-09	5.9E-10	8.6E-01	9.0E-02
V5TG	1.8E-05	1.6E-07	6.1E-02	1.7E-02	3.8E-01	7.3E-01
V1CH	3.9E-24	2.0E-43	2.0E-13	4.0E-20	7.3E-01	6.0E-01
V2CH	2.8E-23	1.6E-38	2.2E-09	2.4E-12	4.2E-01	1.5E-01
V3CH	7.0E-27	4.7E-44	2.2E-14	8.7E-17	6.3E-01	6.8E-02
V4CH	1.6E-17	5.9E-27	4.3E-10	3.3E-11	1.8E-01	1.1E-01
V5CH	3.3E-01	3.7E-02	7.0E-02	5.8E-02	2.9E-01	8.1E-01
V1FC	1.1E-25	1.5E-44	2.8E-12	1.0E-17	8.1E-01	3.8E-01
V2FC	8.4E-30	3.1E-49	5.8E-13	1.1E-17	7.8E-01	1.8E-01
V3FC	9.9E-31	2.7E-52	2.0E-15	2.2E-19	9.2E-01	1.2E-01
V4FC	1.1E-23	4.6E-38	2.8E-15	1.3E-17	1.9E-01	1.6E-01
V5FC	9.2E-09	1.7E-17	1.0E-06	1.6E-09	7.9E-01	8.5E-01
V1PL	2.0E-25	4.8E-42	1.0E-11	2.1E-17	3.4E-01	4.3E-01
V2PL	7.7E-22	1.2E-29	1.4E-07	2.9E-09	8.3E-01	7.3E-02
V3PL	1.3E-26	1.0E-38	1.2E-12	4.7E-15	9.4E-01	5.8E-02

Supplementary Table 12 continued

	GlycA	GlycA sum	GlycB	GlycB Sum	SPC sum	SPC total/GlycA
V4PL	6.1E-17	2.1E-24	3.6E-09	5.0E-10	5.5E-01	1.2E-01
V5PL	2.8E-03	4.8E-05	9.8E-01	8.4E-01	7.2E-01	7.8E-01
**LDL Content**						
LDPN	9.3E-01	3.9E-01	8.2E-01	9.8E-01	1.8E-09	2.7E-01
LDTG	5.3E-08	2.1E-11	2.6E-07	2.3E-07	1.9E-02	4.3E-02
LDCH	3.6E-02	1.6E-01	3.1E-01	1.5E-01	1.5E-12	5.7E-01
LDFC	1.3E-02	7.6E-02	4.2E-01	1.5E-01	6.1E-15	5.9E-01
LDPL	1.4E-02	6.8E-02	1.6E-01	5.2E-02	3.7E-13	5.1E-01
LDAB	9.3E-01	3.9E-01	8.2E-01	9.8E-01	1.8E-09	2.7E-01
L1TG	5.5E-09	9.6E-14	1.6E-06	2.2E-07	2.9E-02	1.5E-01
L2TG	6.7E-01	6.3E-01	6.0E-02	1.3E-01	2.7E-05	2.1E-01
L3TG	1.7E-02	8.6E-02	9.8E-02	5.6E-02	7.1E-06	7.0E-01
L4TG	3.8E-01	1.9E-01	2.6E-01	6.4E-01	3.5E-01	3.3E-01
L5TG	8.4E-06	1.7E-08	3.7E-04	3.2E-04	5.8E-01	6.7E-02
L6TG	3.0E-08	1.6E-08	3.0E-05	3.5E-06	7.2E-01	1.5E-03

	GlycA	GlycA sum	GlycB	GlycB Sum	SPC sum	SPC total/GlycA
L1CH	8.1E-01	3.7E-01	2.9E-01	5.1E-01	1.4E-17	5.2E-01
L2CH	3.0E-10	3.1E-10	3.6E-05	2.8E-06	7.1E-08	4.2E-01
L3CH	9.1E-06	9.7E-06	2.8E-02	3.2E-03	1.5E-10	7.6E-01
L4CH	1.1E-01	3.3E-01	4.5E-01	1.8E-01	1.4E-04	8.8E-01
L5CH	4.4E-01	7.7E-02	8.3E-01	7.8E-01	9.7E-02	4.5E-01
L6CH	6.4E-06	1.4E-07	6.6E-04	3.6E-05	1.4E-01	2.6E-02
L1FC	8.4E-01	2.5E-01	7.7E-02	1.9E-01	1.4E-21	4.5E-01
L2FC	7.5E-07	3.0E-07	5.6E-03	1.9E-03	3.7E-10	4.5E-01
L3FC	3.7E-07	9.9E-08	6.7E-03	4.8E-04	7.2E-12	6.5E-01
L4FC	2.2E-02	3.8E-02	3.5E-01	9.7E-02	2.9E-07	9.2E-01
L5FC	9.5E-01	3.5E-01	8.1E-01	9.9E-01	6.3E-03	4.2E-01
L6FC	2.8E-02	2.1E-02	1.1E-01	8.0E-02	8.0E-03	6.5E-02
L1PL	9.7E-01	3.1E-01	2.6E-01	4.7E-01	1.9E-16	4.6E-01
L2PL	3.4E-10	1.5E-10	3.2E-05	2.0E-06	2.3E-08	4.8E-01
L3PL	1.9E-06	1.5E-06	1.2E-02	9.0E-04	2.5E-10	7.6E-01
L4PL	9.6E-02	2.9E-01	4.0E-01	1.4E-01	1.3E-04	9.4E-01
L5PL	7.1E-01	2.2E-01	7.6E-01	7.8E-01	1.2E-01	4.8E-01
L6PL	3.1E-05	7.8E-07	1.4E-03	1.1E-04	3.7E-02	2.8E-02
L1AB	5.1E-01	8.5E-02	6.0E-02	1.3E-01	2.0E-14	3.5E-01
L2AB	3.4E-09	4.8E-09	5.7E-05	4.0E-06	1.2E-07	4.6E-01
L3AB	4.6E-05	4.2E-05	6.5E-02	7.3E-03	2.1E-09	8.7E-01
L4AB	5.1E-01	9.0E-01	9.6E-01	6.2E-01	3.7E-04	9.7E-01
L5AB	6.8E-02	3.3E-03	2.4E-01	2.2E-01	1.4E-01	2.8E-01
L6AB	6.7E-09	1.5E-12	1.4E-05	1.8E-07	2.5E-01	3.1E-02

Supplementary Table 12 continued

	GlycA	GlycA sum	GlycB	GlycB Sum	SPC sum	SPC total/GlycA
	HDL Content					
HDTG	2.3E-06	3.7E-11	2.5E-04	1.0E-04	2.8E-04	4.3E-01
HDCH	3.4E-05	6.6E-05	5.8E-03	5.9E-03	2.4E-23	7.3E-01
HDFC	2.1E-04	5.5E-04	1.3E-02	1.3E-02	1.1E-26	5.3E-01
HDPL	1.2E-03	8.2E-03	3.2E-02	2.6E-02	1.1E-37	6.5E-01
HDA1	1.1E-02	1.3E-01	8.9E-02	1.3E-01	6.0E-35	6.7E-01
HDA2	2.0E-02	8.2E-05	6.1E-03	3.0E-04	3.8E-18	8.9E-01
H1TG	2.0E-02	3.1E-04	1.6E-03	2.0E-03	2.9E-08	3.0E-01
H2TG	5.0E-07	2.3E-10	1.4E-05	9.2E-06	1.3E-03	6.7E-01
H3TG	7.1E-12	1.8E-18	2.5E-06	7.3E-08	1.1E-01	8.7E-01
H4TG	1.6E-07	2.4E-13	1.9E-02	2.7E-03	8.1E-01	5.0E-01
H1CH	2.4E-03	1.3E-03	3.3E-01	2.7E-01	3.1E-16	8.0E-01
H2CH	3.4E-03	3.3E-03	2.0E-01	1.7E-01	6.8E-22	4.4E-01
H3CH	5.9E-01	8.7E-01	9.2E-01	6.6E-01	8.2E-26	3.3E-01
H4CH	3.2E-03	1.1E-02	8.1E-05	1.5E-04	5.2E-04	9.4E-01
H1FC	3.3E-03	6.6E-03	5.9E-01	5.2E-01	1.6E-23	9.5E-01
H2FC	5.5E-01	7.4E-01	3.3E-02	5.1E-02	9.6E-36	4.7E-01
H3FC	4.5E-01	8.6E-01	9.2E-01	8.4E-01	2.6E-35	4.2E-01
H4FC	2.9E-01	5.9E-01	8.3E-02	5.2E-02	2.4E-07	9.8E-01
H1PL	4.0E-03	5.1E-03	4.4E-01	3.4E-01	4.8E-19	9.3E-01
H2PL	6.2E-02	1.2E-01	6.2E-01	6.7E-01	2.1E-26	3.5E-01
H3PL	9.4E-01	4.5E-01	8.6E-01	5.5E-01	2.8E-27	2.8E-01
H4PL	4.6E-02	2.3E-01	2.9E-04	5.5E-04	1.6E-05	8.5E-01
H1A1	5.7E-04	1.9E-03	1.9E-01	1.4E-01	1.2E-17	8.5E-01
H2A1	3.1E-02	2.3E-01	4.3E-01	7.6E-01	2.4E-29	5.4E-01
H3A1	2.8E-01	3.6E-02	2.5E-01	5.2E-02	3.3E-23	2.7E-01
H4A1	1.7E-01	6.4E-01	8.6E-03	1.8E-02	5.9E-05	9.3E-01
H1A2	5.3E-01	8.1E-01	1.8E-01	1.1E-01	1.2E-22	9.7E-01
H2A2	2.4E-04	9.0E-07	9.6E-09	3.8E-11	6.5E-20	9.3E-01
H3A2	4.5E-08	6.0E-12	2.7E-08	6.3E-12	3.5E-15	5.4E-01
H4A2	4.1E-01	4.3E-02	9.0E-01	6.7E-01	2.6E-03	8.6E-01

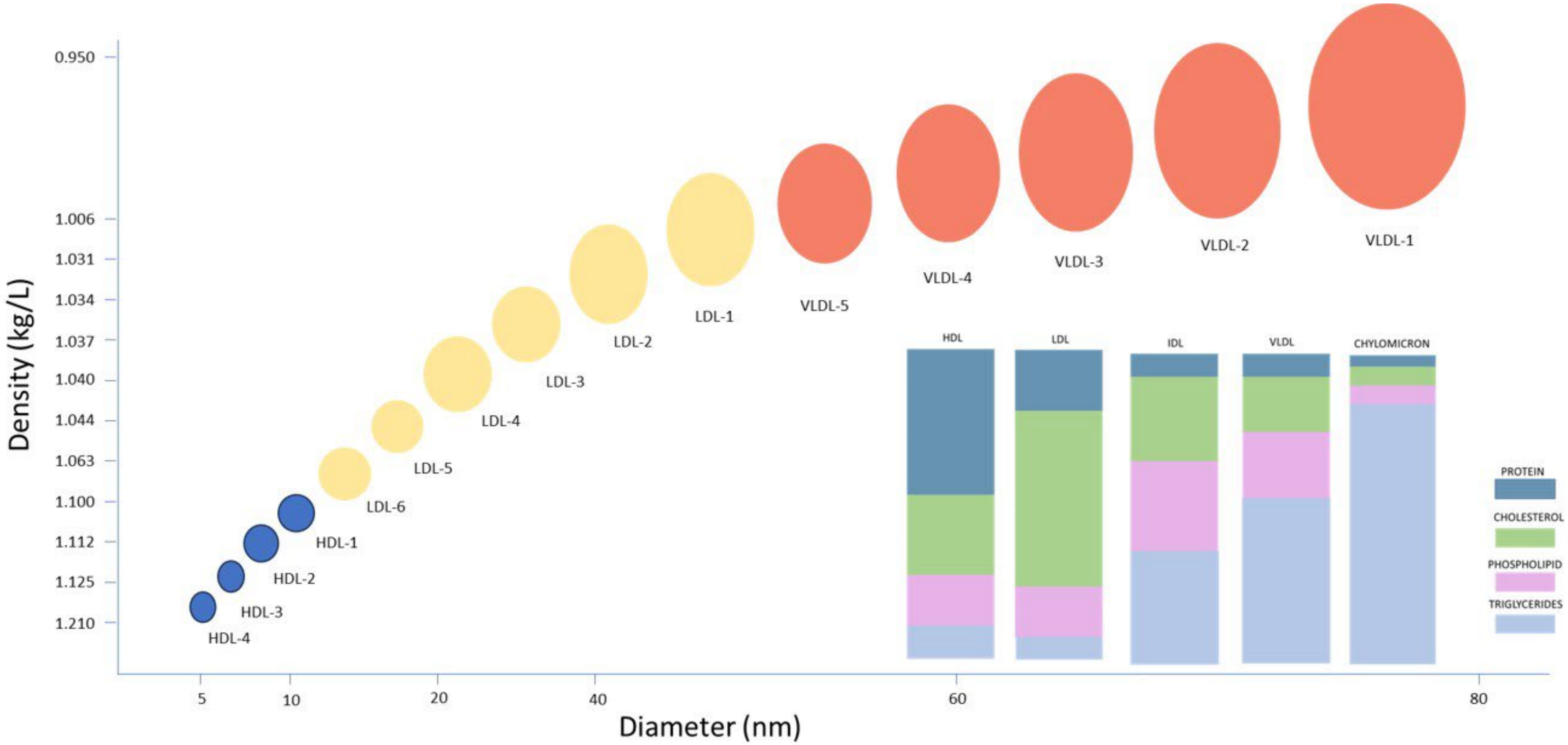

Supplementary Figure 1: Indicative diagram of lipoprotein particle size, density and lipid contents.

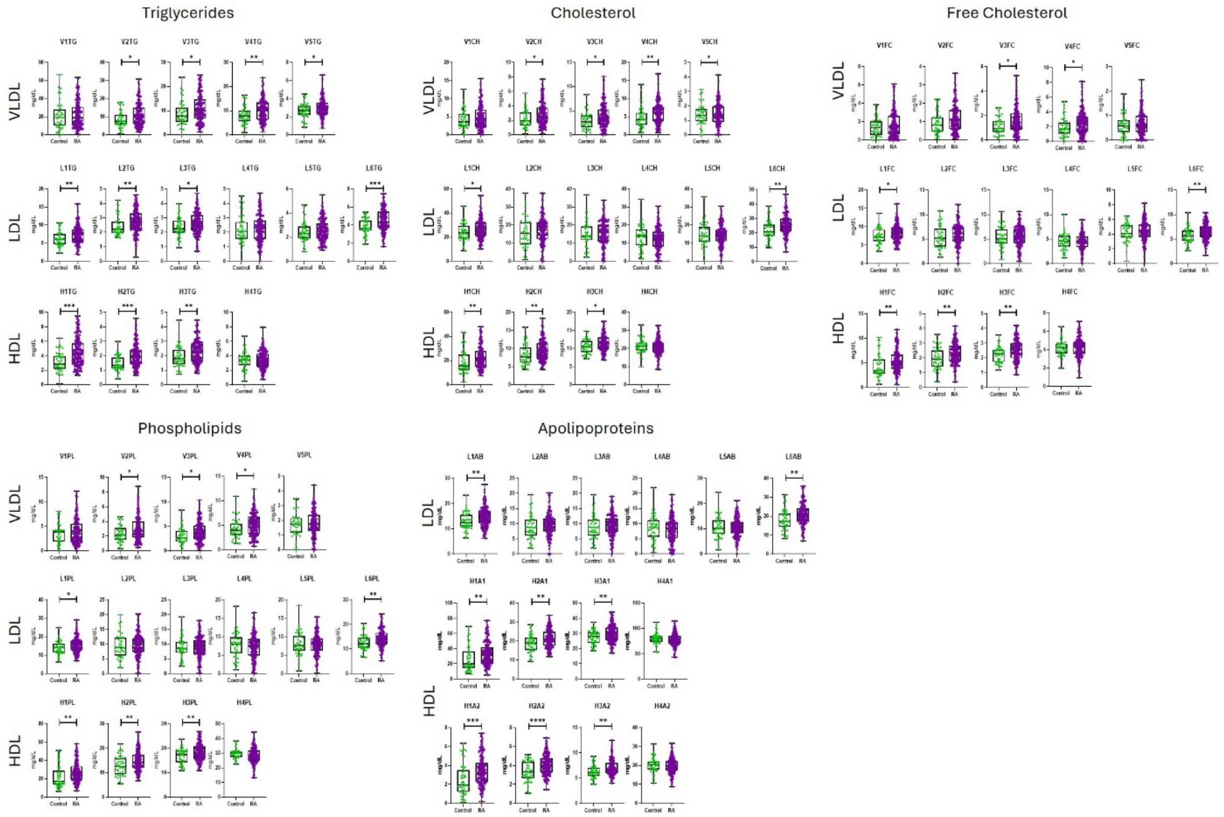

Supplementary Figure 2. Boxplots show the distribution of lipoprotein subfraction concentrations and statistical significance (*p*- value) in RA and control, presenting the mean, standard deviation (SD) and minimum and maximum points The statistical significance is determined by unpaired two-tailed t-test/Mann-Whitney test. Significance is demonstrated with asterisks (*). All lipoprotein species are measured in mg/dL, except particle number (PN), which are measured in nmol/ L.

*$p<0.05$, **$p<0.01$, ***$p<0.001$

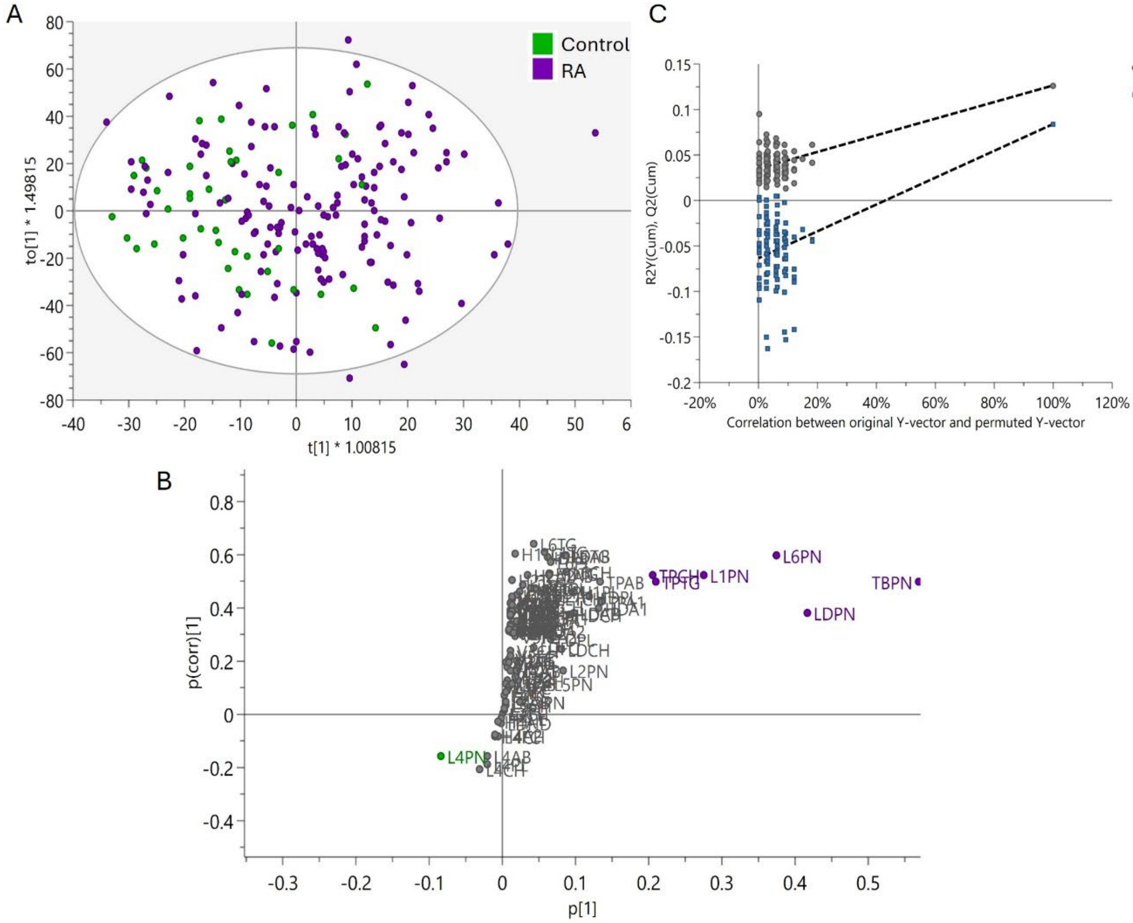

Supplementary Figure 3: OPLS-DA of IVDr lipoproteins and their subfractions in plasma of RA (purple) and Control (green) individuals. A) OPLS-DA scores plot showing the discrimination of the lipoprotein profiles of the two groups (R2X(cum)=0.759; (R2Y(cum)=0.127; Q2(cum)=0.084); B) S-plot demonstrates the lipoprotein features significantly elevated in Control (bottom, left) and RA population (top, right), which are responsible for the clustering (VIP>1.5). The features in grey, located centrally, do not significantly impact the separation. C) Permutation testing with 100 random permutations confirms the validity and reliability of the OPLS-DA model.

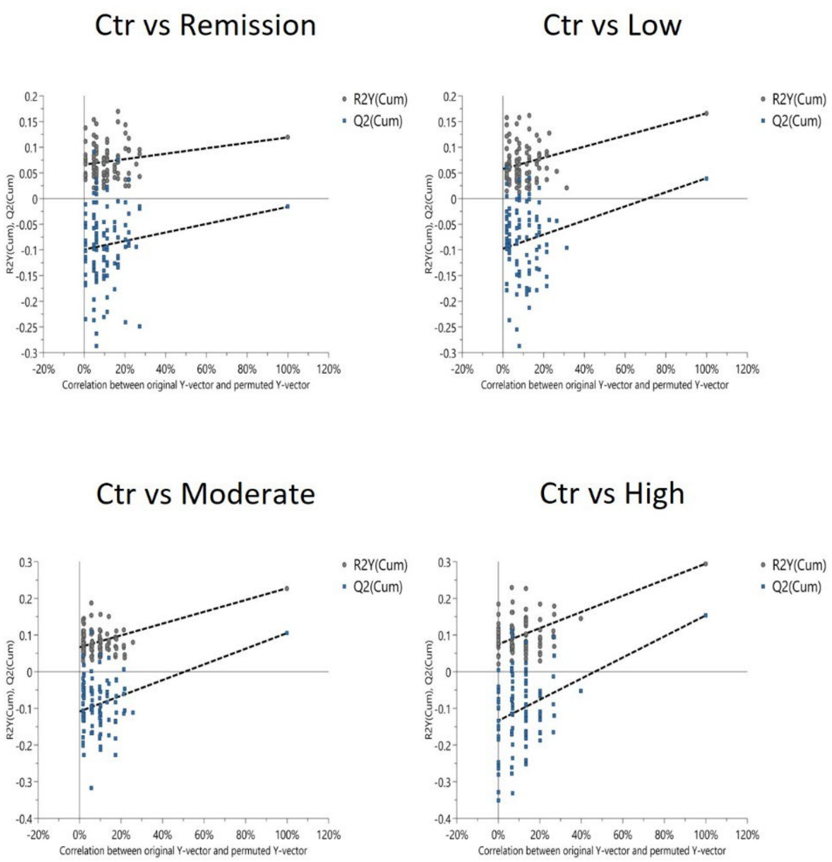

Supplementary Figure 4: Permutation test with 100 random permutation evaluates the validity and reliability of the OPLS-DA models comparing the lipoprotein profile of DAS28-classified RA subgroups with controls.

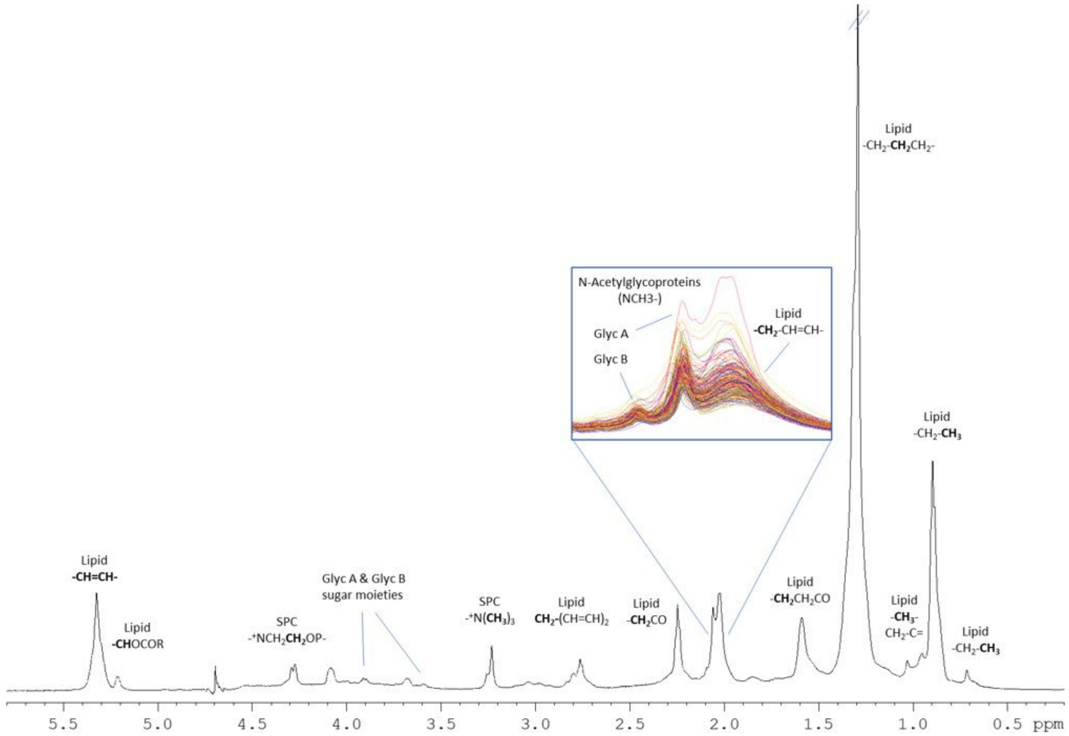

Supplementary Figure 5. A representative NMR LED spectrum (0.5 – 5.5 ppm) with annotation of the corresponding peaks. In the inset, the region of the acetyl group resonance (2.15-1.95 ppm) of all LED spectra (161 RA patients) is shown.

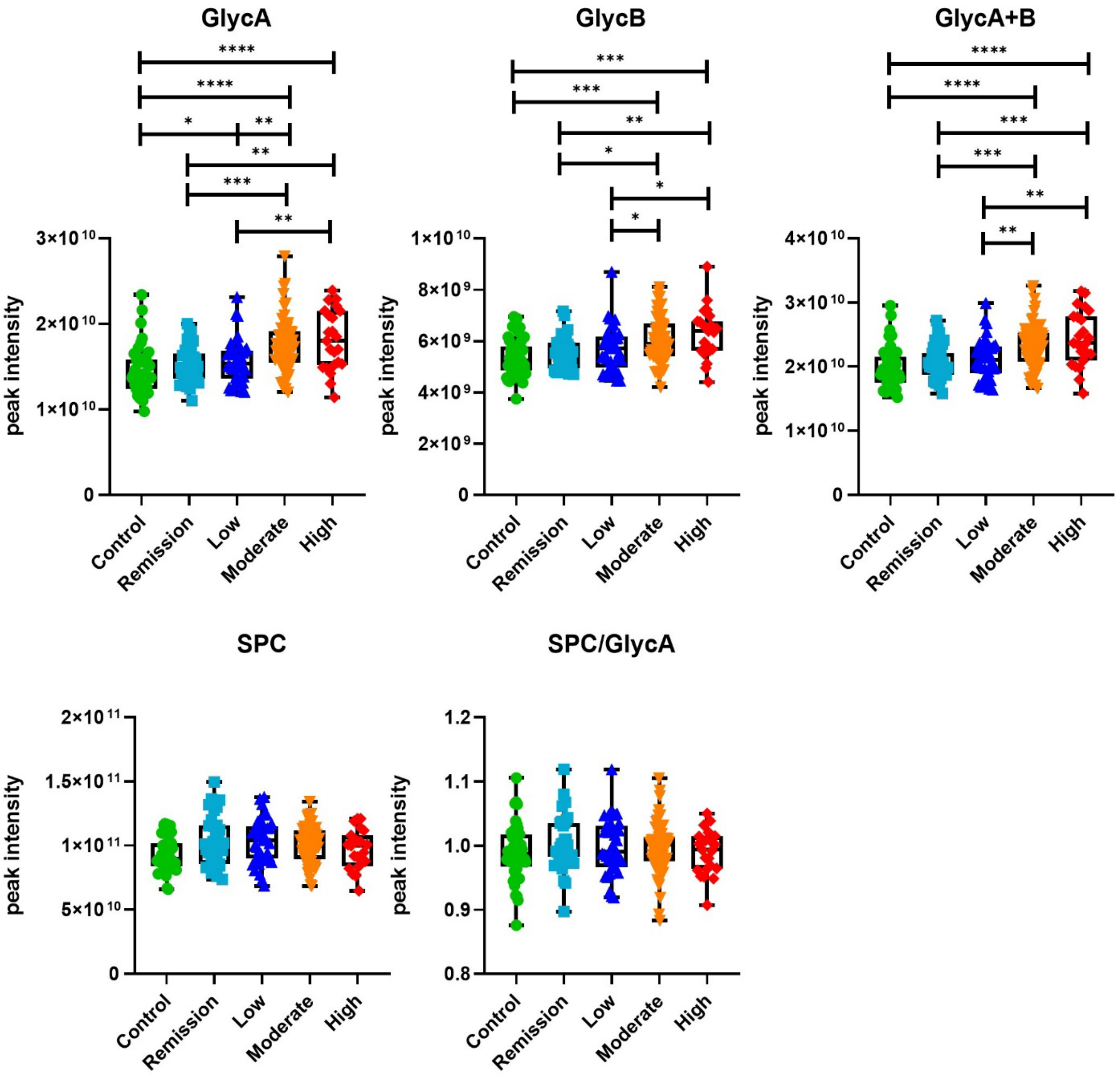

Supplementary Figure 6: Changes in glycoprotein and SPC levels in RA DAS28-classified subgroups, namely Remission (blue), Low (dark blue), Moderate (orange) and High (red), compared to controls (green). Statistical significance between the groups is calculated with one-way ANOVA/Kruskal-Wallis test and corrected for multiple comparisons. Statistically significant comparisons are indicated with asterisks (*).

*p<0.05, **p<0.01, ***p<0.001, ***p<0.0001

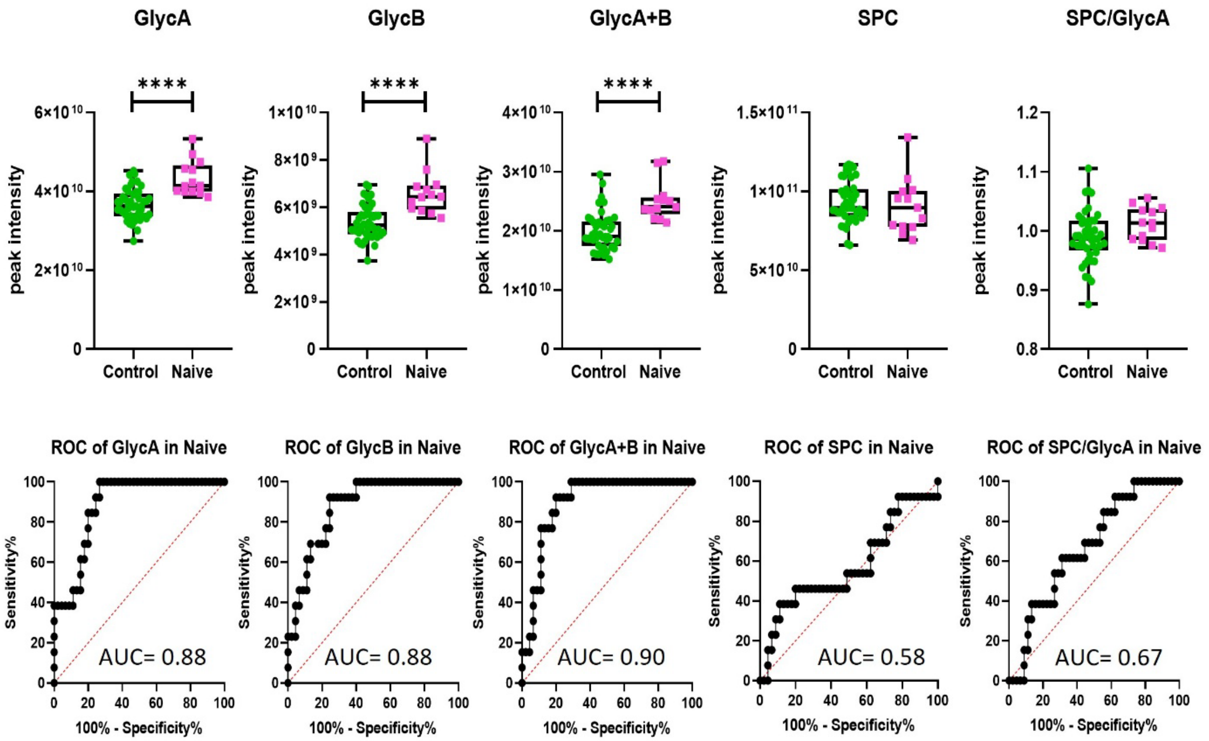

Supplementary Figure 7: Changes in glycoproteins and SPC signal levels in DMARD-Naive RA population compared to controls. A) Boxplots comparing intensities of the markers between the investigated groups, B) ROC curves showing the predictive capacity of each marker with the respective AUC. The statistical significance is determined by unpaired two-tailed t-test/Mann-Whitney test. Significance is demonstrated with asterisks (*).

*p<0.05, **p<0.01, ***p<0.001

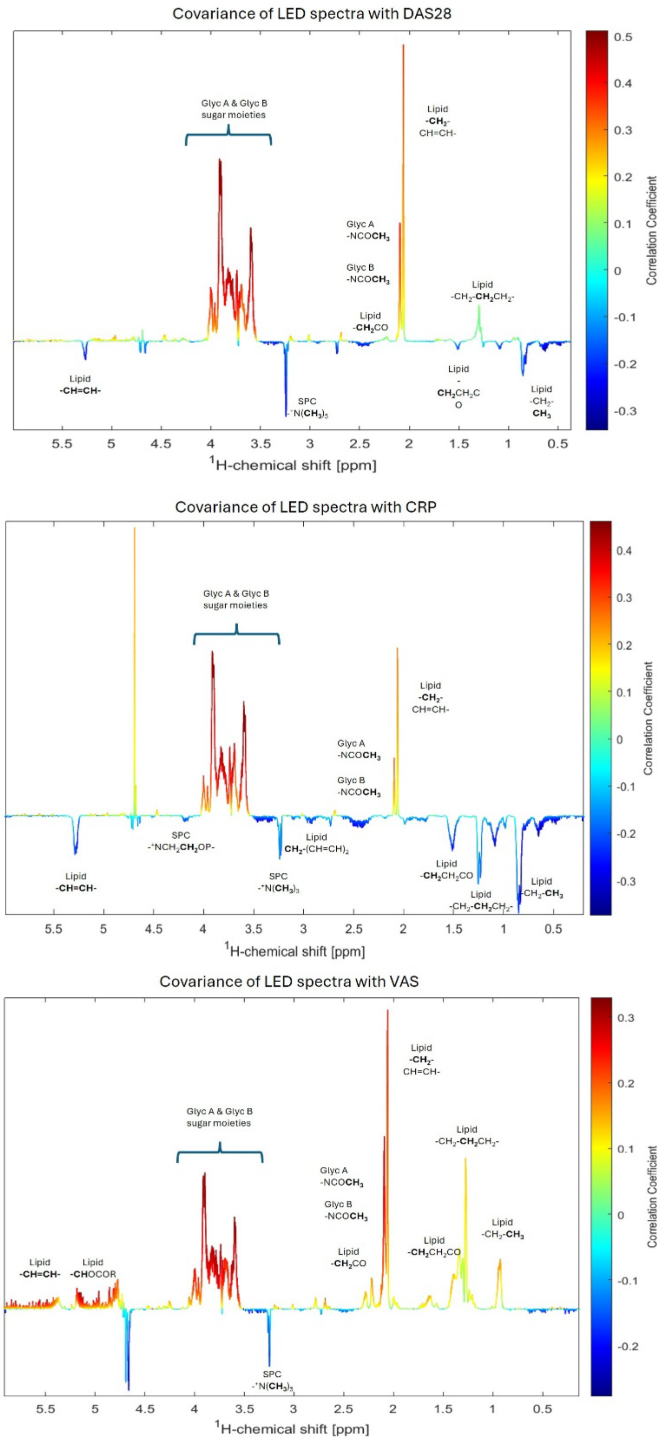

Supplementary Figure 8: NMR pseudo-spectra demonstrate the covariance of the 161 NMR LED spectra, containing 1040 features (lipid signals) with clinical parameters, A) DAS28, B) CRP, and C) VAS. The correlation coefficient indicates positive (red) and inverse (blue) association between the spectral peaks and the clinical parameters.

A

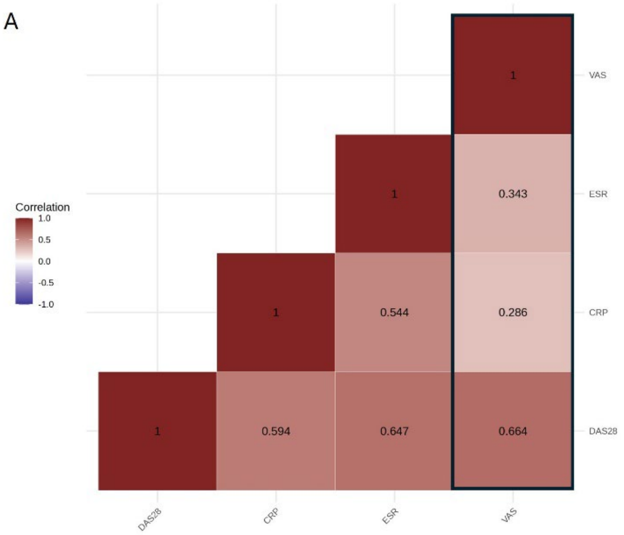

B

	r^2	***p*-value**
**L6TG**	0.62	1.10E-03
**H1TG**	0.59	1.70E-03
**H4A2**	0.46	9.60E-03
**H4CH**	0.41	1.48E-02
**H4PL**	0.37	2.38E-02
**HDTG**	0.56	3.82E-02
**H2TG**	0.29	4.84E-02

Supplementary Figure 9: A) Correlation between studied markers of RA monitoring DAS28, CRP, ESR, and VAS. B) The association of VAS with individual lipoprotein species demonstrating the highest correlation (r-corr) with VAS in DMARD-Naïve RA patients. The statistical significance of each correlation was assessed via the t-distribution, and the *p*-value is presented.
